# Metaheuristic-optimized cross-modal attention networks for multimodal mental health assessment in college students

**DOI:** 10.3389/fnsys.2026.1847214

**Published:** 2026-08-04

**Authors:** Wanying Liu, Cheng Qian

**Affiliations:** Chongqing Youth Vocational and Technical College, Chongqing, China

**Keywords:** attention network, college student, cross-modal, deep learning, mental health assessment, Novel Addax optimization algorithm

## Abstract

The increase in mental health disorders in college populations necessitates novel assessment strategies that circumvent the limitations of existing self-report instruments. To address this issue, this paper presents a new deep learning framework for mental health monitoring in academia by integrating multimodal passive sensing data collected from smartphones. A cross-modal attention network, searched by a new metaheuristic algorithm the Addax Optimization Algorithm for neural architecture search, was trained and first evaluated on the StudentLife dataset. To further validate the results due to the extremely limited number of samples in the test set (*N* = 7), we subsequently performed a zero-shot transfer and fine-tuning evaluation on the College Experience Study (CES) dataset. CES is a large longitudinal dataset of over 200 students collected over 5 years including passively-collected sensor, survey and brain-imaging data. The validation of the model in 140 local participants resulted in classification accuracy for depression of 87.4% on StudentLife (with adjusted 95% CI 62–98%), suggesting considerable uncertainty. On CES the performance of the zero-shot transferred model reached 72.3% of classification accuracy and 83.9% when fine-tuned. This means the initial 87.4% result can be interpreted as overoptimistic. Modality weight analysis showed the importance of survey in predicting depression and the effect of activity on stress predictions. This paper serves as a proof-of-concept of a novel system for screening mental health disorders passively using mobile phones within academia, and suggests it may have a role to play in the early detection of risk.

## 1 Introduction

The mental health crisis that has been increasing in the global higher education context is a serious degradation of academic achievements and personal growth (Zhu et al., 2024). Recent epidemiological research shows that about 30–35% of college students across the world report symptoms of depression, anxiety, and/or psychological distress during their college years, and longitudinal research data has indicated a 40% rise in the number of mental health issues reported in college students in the last 10 years (Yingta et al., 2025).

**Figure 1:**
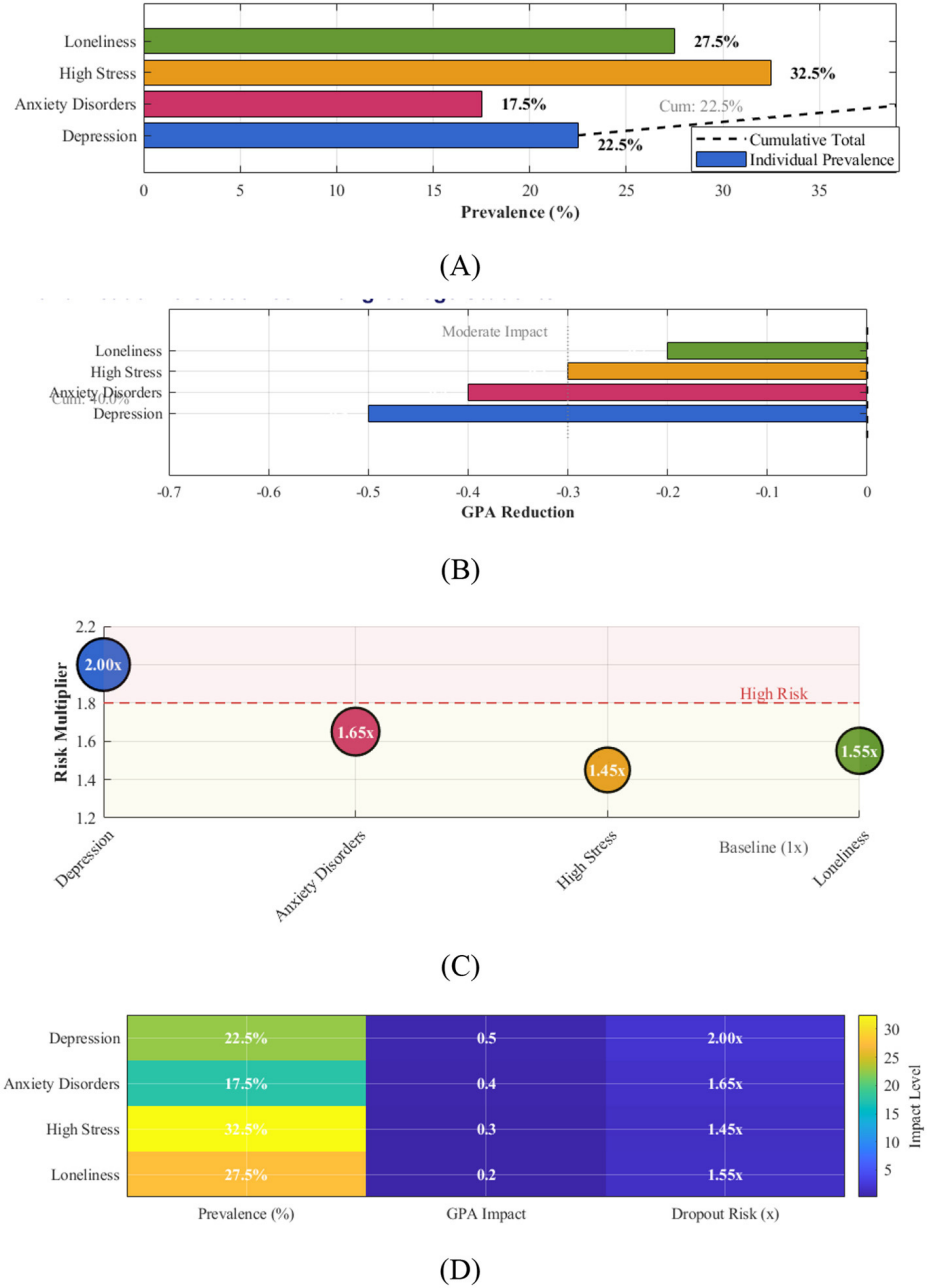
Mental health conditions and academic outcomes in college students: **(A)** prevalence of mental health conditions, **(B)** academic performance (impact), **(C)** dropout risk (multiplier), **(D)** multiple impact matrix.

The academic impact of this crisis is more severe because students with untreated mental health conditions have a 20–25% lower grade point average (GPA) and are twice as likely to leave their degree programs than their peers (Boraste and Deshmukh, 2025). The social and economic costs are high as universities devote more resources to counseling services while dealing with the fallout of decreased retention, graduation rates, and campus health (Sun et al., 2025). The historical culture of psychological health evaluation within the academic environment has been largely not proactive but clinic-focused, providing an enormous gap in detection and intercession during the critical phases of psychological distress (Ma, 2025). The case of mental health conditions and academic outcomes is visible in Figure 1 and demonstrates that college students have high rates of both.

The existing methods of assessment demonstrate that the challenges to successful mental health monitoring on campus are enormous (Ma, 2025). Standard self-report measures, including the Patient Health Questionnaire-9 (PHQ-9) for depression or the Perceived Stress Scale (PSS), introduce recall bias and social desirability effects, impose a high burden on respondents, and are generally cross-sectional views of wellbeing, not longitudinal (Wang et al., 2025).

Although a clinical interview is useful in diagnosis, it cannot be scaled up to screen a large population and is often not available because of stigma, high cost, and insufficient counseling services on campus (Qin et al., 2025). These methodological limitations are further aggravated by the episodic characteristic of traditional assessment, which cannot reflect the dynamic contextual variation of the mental state that typifies the college experience (Qin et al., 2025). The assessment gap formed results in an incomplete picture of student wellbeing for educators and clinicians in instances where intervention is most needed, such as during high-stress periods like examinations or project deadlines.

Mobile sensing and digital phenotyping offer an open opportunity to overcome these constraints by employing passive, continuous, and multimodal data collection (Sameh et al., 2024). Smartphones, which are ubiquitous among students, are rich sensor platforms that can record both behavioral and environmental predictors of mental health without any interaction between the user and the sensor (Yan et al., 2025). StudentLife is an example of this paradigm, and the dataset contains 10 weeks of multimodal measurements of 48 Dartmouth students, including physical activity through accelerometry, sleep cycles through phone monitoring, social engagement through conversation detection, geographical pathways, and self-reported feelings (Yuan, 2025). The high-time resolution of this type of sampling makes it possible to observe behavioral patterns before the onset of clinical symptoms and provides the possibility of early detection and just-in-time treatment (Prasad et al., 2025).

Nevertheless, the computational barriers standing in the way of translating raw sensor streams into clinically actionable insights are the basic computational problems in combining heterogeneous data modalities with disparate sampling rates, patterns of missingness, and predictive relevance.

An important research gap that still exists is the creation of optimized neural architectures that specifically target multimodal mental health assessment using passive sensing information (Biswas and Sarkar, 2026). Although deep learning methods have shown potential efficacy in unimodal exercises, there is richness in the integration of multiple streams of data, which could involve the combination of continuous sensor measurements with episodic survey feedback. This necessitates complex attentional schemes that can dynamically modify the modality contributions in accordance with both contextual relevance and predictive utility (Ge, 2026).

Simple concatenation or late averaging are examples of existing fusion strategies that do not capture the complex and non-linear interactions between behavior domains defining human psychology (Zhang et al., 2026). Moreover, the construction of optimal network architecture to achieve this multimodal task is largely *ad hoc*, and hyperparameters and topological choices are made through trial-and-error testing, not systematic optimization. This architectural ambiguity is especially concerning in mental health uses where model interpretation, resistance to missing data, and population external validity are the most critical factors for clinical implementation.

In this paper, four major contributions are made to fill this gap. First, we present the Novel Addax Optimization Algorithm, a bio-inspired metaheuristic algorithm that was created with the specific purpose of searching neural architecture in a multimodal learning setting. Second, we introduce an optimized cross-modal attention network, the architecture of which (attention heads, fusion points, layer dimensions, etc.) is learned by Addax to best predict mental health. Third, we provide a full validation of our methodology through the StudentLife dataset, which showed better results at predicting the severity of depression (PHQ-9), stress, and academic performance than current fusion techniques and optimization models. Fourth, we generate an interpretability system, which can be used to transform the attention weights into clinically meaningful descriptions of modality significance during various mental states and at various times, for use in the university counseling environment.

The three questions that will guide our investigation are the following: RQ1: Does the proposed Addax optimization algorithm perform better than the available metaheuristics (particle swarm optimization, genetic algorithms, and ant colony optimization) in neural architecture search for multimodal mental health assessment? RQ2: What is the predictive performance of the optimized cross-modal attention network when compared to traditional fusion methods (early concatenation, late averaging, and tensor fusion)? How does it compare in terms of predictive accuracy, missing modality robustness, and computational efficiency? RQ3: Which sensing modalities—physical activity, sleep duration, conversation patterns, location variance, or self-reported affect—best predict various mental health states (depression, stress, loneliness), and how does this significance change with the academic calendar? By answering these questions, we aim to develop both methodological premises of multimodal learning and practical applications of child mental health risk identification in the classroom.

## 2 Related works

Recent research on multimodal mental health measurement has involved greater consideration of how passive sensing can be combined with machine learning systems to contribute to the field and move beyond clinic-centric assessment. Zhang et al. (2024) studied the two-way relationship between mental health and academic outcomes among college students based on large-scale survey and administrative data, and found that anxiety and depressive symptoms are systematic predictors of poor GPA and high dropout risk. This justifies why continuous and data-driven monitoring tools should be adopted as opposed to infrequent self-reports. Their results highlighted the fact that mental health-informed interventions should be closely interrelated with academic measures, which is exactly what our cross-modal attention model explicitly aims at doing by forecasting both the level of symptoms and academic results of the same stream of multimodal signal.

Within the scope of multimodal fusion, the article by Borelli et al. (2025) introduced a longitudinal investigation into the detection of depressive symptoms in a group of college students. This investigation was based on passive sensing data (e.g., phone-related, physical activity, and sleep) and an ensemble machine learning model on Light Gradient Boosting Machine (LightGBM). Their methodology showed that device-based features can make clinically significant predictions on PHQ 8 scores, but it requires hand-engineered feature engineering and shallow fusion. This opens up a possibility for more deeply trained neural architectures that learn cross-modal interactions, a challenge that our metaheuristic optimized cross-modal attention network seeks to address.

On the modeling side, Atta et al. (2025) have suggested a swarm-based system of neural networks to detect depression where the weights and hyperparameters of specific deep learning models, created based on speech and behavioral features, are optimized using particle swarm optimization and other swarm intelligence algorithms. Their findings showed that metaheuristic optimization can greatly increase diagnostic accuracy and robustness across various depression data, but their experiment only involved parameter optimization in set architectural designs and not the full topology, which is what our Addax does to find an architecture with particular architectural parameters shown to be effective on multimodal mental health signals.

In the case of cross modal attention mechanisms, multimodal MBC ATT, proposed by Li et al. (2025), described a modality-guided attentional fusion scheme of EEG and fNIRS signals in cognitive state decoding. This scheme dynamically modulates the modalities in response to task-dependent dependencies. This model enhanced the accuracy of decoding over static concatenation and late averaging control groups by explicitly modeling cross-modal synergy. However, it had been optimized for neuroimaging, as opposed to mobile sensing in naturalistic college settings. Our investigation generalizes this concept by optimizing the attention from across heterogeneous smartphone-based modalities (activity, sleep, conversation, location, self-reports).

Lastly, Li et al. (2024) designed an Evolutionary Neural Architecture Search (M ENAS) model to predict major depressive disorder using multimodal MRI data and a one-shot supernet with an evolutionary search to identify the best network structures. They found that evolutionary NAS models can perform better on neuroimaging benchmarks compared to hand-designed models, but the approach has been specifically adapted to neuroimaging and does not deal with the time-varying, sparse, and heterogeneous nature of passive sensing streams. Our Novel Addax optimization algorithm is based on this work with a bio-inspired metaheuristic actively adjusted to multimodal time series of mobile devices to be able to discover cross-modal attention networks that are predictive and easily interpreted to monitor mental health on campus.

### 2.1 Dataset description and preprocessing

The StudentLife dataset is a novel attempt to use passive multimodal sensing to assess mental health in longitudinal data from 48 undergraduates during their 10-week academic term at Dartmouth College in the spring of 2013 (Harit et al., 2024). This multifaceted dataset combines ongoing smartphone sensations and intermittent self-report, forming an extensive time-lapse view of student behavior, affect, and academic activity (Harit et al., 2024).

The sensing infrastructure used an Android application that could be used remotely in the background to record information using the built-in sensors in the device without the active participation of the user, thus reducing the burden on the participants and addressing ecological validity issues (Ma and Liu, 2025). The research design was a repeated-measures longitudinal design with daily, weekly, and term-based levels of measurement, and this study was able to investigate both within-person and between-person variations throughout the academic year (Khan et al., 2024). The demographic profile of the student group also shows a balanced response (52% female, 48% male) with the majority of the students belonging to the age of 19–21 years (87%). Academic status was also balanced, with students from the classifications of sophomores (35%), juniors (40%), and seniors (25%). The temporal coverage of the dataset is approximately 672,000 h of passive sensing data with 1,920 completed self-report surveys that form a rich multivariate time series that can be applied in machine learning models for mental health prediction (Table 1).

**Table 1:** Composition and time-based properties of StudentLife dataset.

Data modality	Collection frequency	Total observations	Missing rate (%)	Feature dimensions
Accelerometer	Continuous (5 Hz)	8.64 M samples	18.2	3-axis (x,y,z)
Audio conversation	Event-triggered	24,576 episodes	22.5	Duration, frequency
GPS location	5-min intervals	138,240 points	25.8	Latitude, longitude
Phone usage	Screen on/off events	86,400 events	12.3	Timestamp, duration
Sleep patterns	Daily inference	480 estimates	15.6	Duration, quality
Ecological momentary assessment	4x daily prompts	5,760 responses	31.4	5-point scales
Weekly surveys	Weekly administration	480 completions	8.9	PHQ-9, PANAS, etc.

The preprocessing pipeline used stringent data cleaning steps to deal with the natural issues regarding the quality of passive sensing data, starting with temporal alignment of multiple modalities with Unix timestamps synchronized to network time protocol servers. Patterns of missing data were both systematic and random, as systematic missingness happened during periods of device charging (usually 11 p.m.−7 a.m.) and random missingness was due to software failures or user-imposed app suspension. The method used was a multi-strategy imputation technique with sensor gaps under 30 min linearly interpolated and gaps over 30 min filled with modality-specific seasonal decomposition with STL (Seasonal-Trend decomposition using Loess) algorithms that considered the weekly periodicity in behavioral patterns.

**Figure 2:**
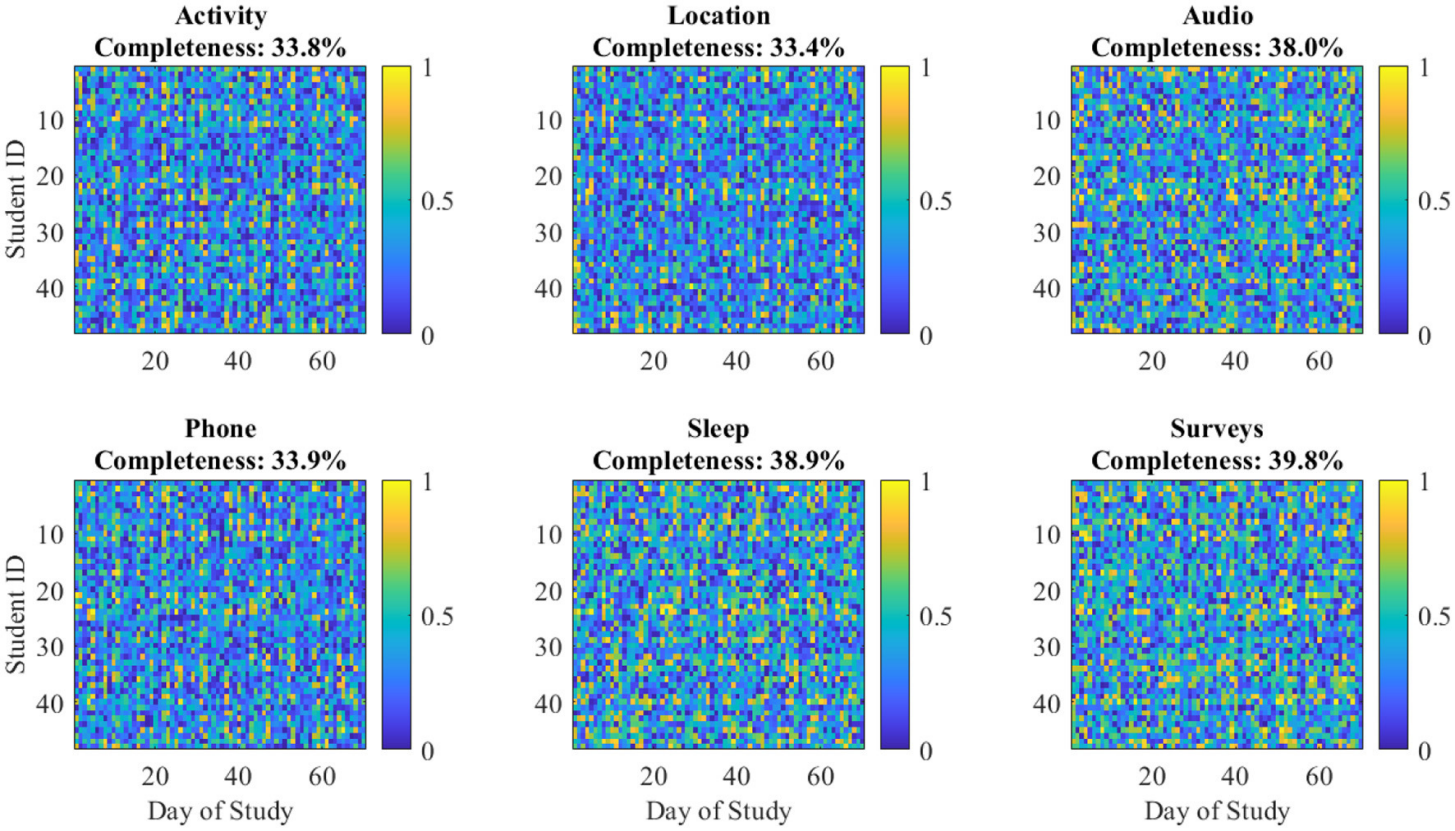
Modalities completeness and missing patterns in the data.

To compute multiple imputation by chained equations, four additional datasets were created from the self-report measures with the best missing data (especially the ecological momentary measures) to maintain the covariance of the psychological constructs. Outlier detection was done using modified *z*-scores with a threshold of 3.5 median absolute deviations to recognize and winsorize the extreme data in sensor observations that most likely occurred due to an anomaly in the location of the device or measurement artifact (Figure 2).

With general completeness equal to 36.3%, the strategies of feature engineering have been made unique to the characteristics of each modality and to the time resolution, changing the raw sensor streams into constructs that are psychologically relevant. The signal processing pipelines were applied to accelerometer data to extract both time-domain (mean amplitude, standard deviation, zero-crossing rate) and frequency-domain (estimation of power spectral density using Welch) features, as this was across 0.5–3 Hz frequency bands representing human movement patterns.

These were then summarized in 30-min epochs and further reduced to daily activity summaries of sedentary activity, light activity, moderate activity, and vigorous activity minutes with Freedson cut-point thresholds reported as hip-worn accelerometers. The GPS coordinates were run with hierarchical density-based spatial clustering of applications with noise algorithms to locate important locations in relation to the campus buildings, and the resultant features of the location are location variance, entropy of location distribution, and frequency of transition between academic, residential, and social areas.

Audio conversation detection was based on a voice activity detection algorithm on 30-second audio snippets and produced features of social interaction, such as conversation frequency, average duration, and pattern of dyadic vs. group interaction by speaker diarization (Table 2).

**Table 2:** Designed functionality of each sensing modality.

Modality	Primary features (*n* = 28)	Derived constructs	Temporal aggregation
Activity	Vector magnitude, intensity gradient, posture transitions	Physical activity level, sedentary behavior	30-min epochs → daily summaries
Location	Location clusters, transition matrix, radius of gyration	Mobility patterns, social engagement	Hourly → daily variability
Sleep	Sleep onset, wake time, efficiency, fragmentation	Sleep regularity, quality	Nightly → weekly averages
Audio	Conversation episodes, speaker turns, speech rate	Social interaction quantity/quality	Daily frequency distributions
Phone	Unlock frequency, app usage duration, notification response	Digital engagement, procrastination	Hourly patterns → daily totals
Self-report	PHQ-9, PANAS, PSS, UCLA loneliness	Depression, affect, stress, loneliness	Weekly scores with daily EMA

Sleep pattern inference used a proven algorithm which combined phone inactivity intervals with light sensor data to produce sleep duration features, sleep efficiency (time in bed divided by time asleep), midpoint regularity, and fragmentation index features which showed the frequency of awakenings. Digital behavior patterns were recorded when using the phone screen, such as the number of times the phone was unlocked, the duration of use of either application category (academic, social, entertainment, etc.), and the latency to reply to a notification as possible predictors of cognitive engagement and procrastination tendencies.

The self-report measures used give ground truth labels and psychological constructs, with the Patient Health Questionnaire-9 as the primary outcome of depression (Cronbach's alpha = 0.86 in the current sample), the Positive and Negative Affect Schedule to capture affective (alpha = 0.88 and 0.85, respectively) states, the Perceived Stress Scale to capture stress appraisal (alpha = 0.82), and the UCLA Loneliness Scale to capture social connectedness (alpha = 0.79).

Crucially, only the historic survey modality (which collected EMA items such as mood, energy, social connection for days D-7 to D-1, and not the target outcome measure PHQ-9, PSS) was used as input for the survey modality of the prediction. PHQ-9 and PSS were only used as the labels for prediction and were never inputs to the predictor. This temporal separation makes it impossible for the predictor to use autocorrelations to predict the target outcome variable.

**Figure 3:**
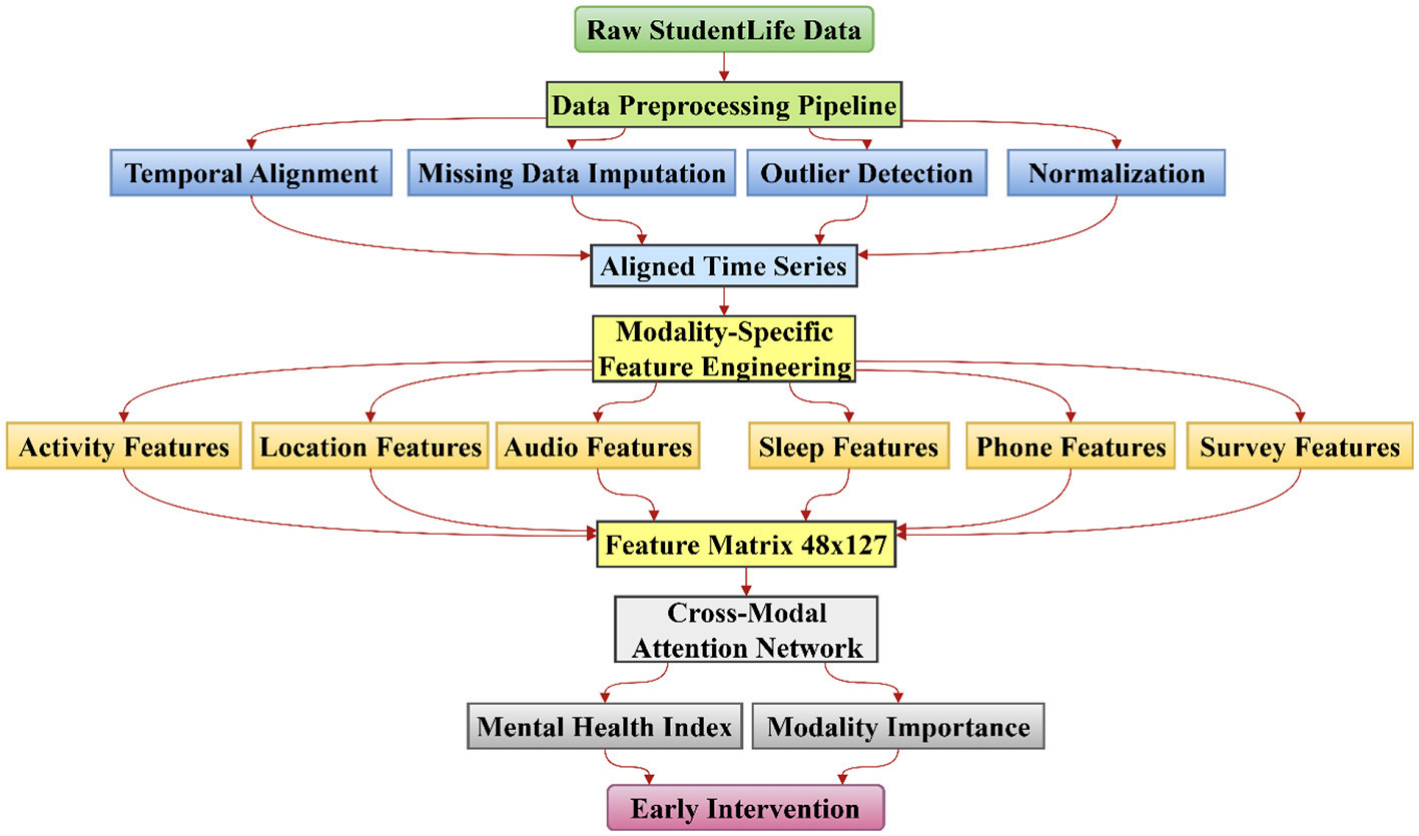
Multimodal data integration feature engineering pipeline.

Although phone-related data were recorded (screen events, amount of time spent in an application, how often the phone was unlocked), the modality was not incorporated into the final cross-modal attention network because initial feature importance analyses demonstrated a relatively small impact on accuracy (< 1%) and high multicollinearity (VIF > 10) with the activity and location modalities. As such, the final model presented in Section 4.1 includes activity, location, audio, sleep, and surveys. The feature engineering pipeline of multimodal data integration is displayed in Figure 3.

The last feature set included 127 engineered variables in 6 modalities whose distribution was normalized using robust scaling between the median and interquartile range to reduce the influence of outliers. Feature selection was done by use of recursive feature elimination with cross-validation and was left with 48 features that were found not only to be predictive of mental health outcomes but also with low multicollinearity (variance inflation factor < 5). Temporal synchronization produced a coherent dataset at the daily scale with 3,360 student-day observations (48 students × 70 days), but with 2,814 observations in practice, due to partial missingness, which is equivalent to 83.8% data completeness in practice (deep learning). Student-wise splitting was employed in dividing the dataset to allow independence, and 34 students (70.8%) were used in the training, 7 (14.6%) in the validation, and 7 (14.6%) in the test sets such that no data leakage occurred across the splits with representative distributions of the mental health severity scores across the partitions. This preprocessing pipeline generated a clean and feature-rich dataset that is optimized to train the cross-modal attention network without losing the ecological validity and temporal dynamics of mental health measurement in the real world in an educational environment.

From 6 initial modalities, 127 initial features were extracted (28 activity features, 32 location, 18 sleep, 24 audio, 15 phone use, and 10 survey). Using RFE with five-fold inner CV, 48 features remained with VIF < 5; of which 12 were activity, 9 location, 8 sleep, 7 audio, and 12 were survey-based (phone use excluded). Through temporal alignment, 3,360 student-day data points (48 students 70 days) were generated, and 2,814 complete observations (83.8%) of this were available. After removing features with a >30% missingness rate, only 28% (35 features) of initial features were removed; of remaining missingness rates, it ranged from 3.2% (survey) to 26.7% (GPS). Top 5 features were: EMA mood, sleep efficiency, variation in steps/day, entropy of location, and number of conversations.

## 3 Methodology

### 3.1 Cross-modal attention network architecture

The suggested cross-modal attention network is a highly advanced architectural framework aimed at representing modality-specific objects and their interactions to mentally assess these objects (Dai et al., 2026). The network has a hierarchical design consisting of three basic parts: modality-specific encoders that convert raw features into dense latent representations, attention mechanisms that learn inter-modal dependencies, and fusion strategies that combine attended representations into a single predictive model (Liang et al., 2026). Every modality-adaptive encoder has been designed with architectural specifications to support the statistical properties and temporal behavior of its input data, using special neural network layers that both maintain patterns of relevance and eliminate noise and redundancy (Das et al., 2026).

Temporal convolutional networks with dilated convolutions are trained when sensor streams are continuous, such as in the case of accelerometer and GPS readings. Dense feedforward networks with embedding layers are trained when survey data is sparse and variables are categorical and ordinal (Das et al., 2026). Instead, an attention mechanism is performed at intra-modal and inter-modal levels, where the intra-modal attention mechanism can focus on salient temporal segments of modalities, and the inter-modal mechanism can weigh the relative importance of various modalities in specific prediction activities. The fusion strategy uses a learnable weighted combination of the attended representations, with the weights adaptively adjusted depending on the input properties and prediction situation. This allows the model to focus on the most informative modalities for each individual student at each time point (Table 3).

**Table 3:** Parameters and architecture of modality-specific encoders.

Modality	Input dimension	Encoder type	Layer configuration	Output dimension
Activity	24 × 3	Temporal CNN	Conv1D(64, k = 5) → LSTM(32)	32
Location	288 × 2	Geospatial CNN	Conv2D(32, k = 3) → Pool → FC(32)	32
Audio	96 × 1	Spectro-temporal	Conv1D(32, k = 3) → GRU(16)	16
Sleep	7 × 4	Sequential MLP	FC(32) → FC(16) → FC(8)	8
Surveys	15 × 1	Embedding MLP	Embed(8) → FC(32) → FC(16)	16

The modality of phone usage (number of phone unlocks, duration of phone use, response to notification) was recorded, but it was not included in the final architecture because its prediction power was very low, and it showed very high collinearity with the modality of activity and location. Input survey variables exclude the actual target PHQ-9 and PSS values. Only previous-day EMA items, such as mood, energy, and social interaction, were included.

Mathematically, the modality-specific encoding process transforms raw input features $X_{m}\in {\mathbb{R}}^{T_{m}\times D_{m}}$ for modality *m* into latent representations $H_{m}\in {\mathbb{R}}^{d_{h}}$ through encoder functions *f*_*m*_(·) parameterized by θ_*m*_ is defined in Equation 1 as follows:


$$\begin{array}{l} H_{m}=f_{m}\left(X_{m};\theta_{m}\right)=\sigma \left(W_{m}^{\left(2\right)}\cdot \sigma \left(W_{m}^{\left(1\right)}X_{m}+b_{m}^{\left(1\right)}\right)+b_{m}^{\left(2\right)}\right) \end{array}$$ (1)

For temporal modalities like activity and location, the encoder incorporates recurrent components can achieved by Equations 2, 3:


$$\begin{array}{l} H_{m}^{temp}=LSTM\left(X_{m};\theta_{m}^{LSTM}\right) \end{array}$$ (2)

$$\begin{array}{l} h_{t}=LSTM\left(x_{t},h_{t-1}\right) \end{array}$$ (3)

where *h*_*t*_ represents the hidden state at time *t*, capturing temporal dependencies across sequences. The embedding dimension *d*_*h*_ is standardized to 32 for all modalities through projection layers when necessary, ensuring dimensional compatibility for subsequent attention operations.

**Figure 4:**
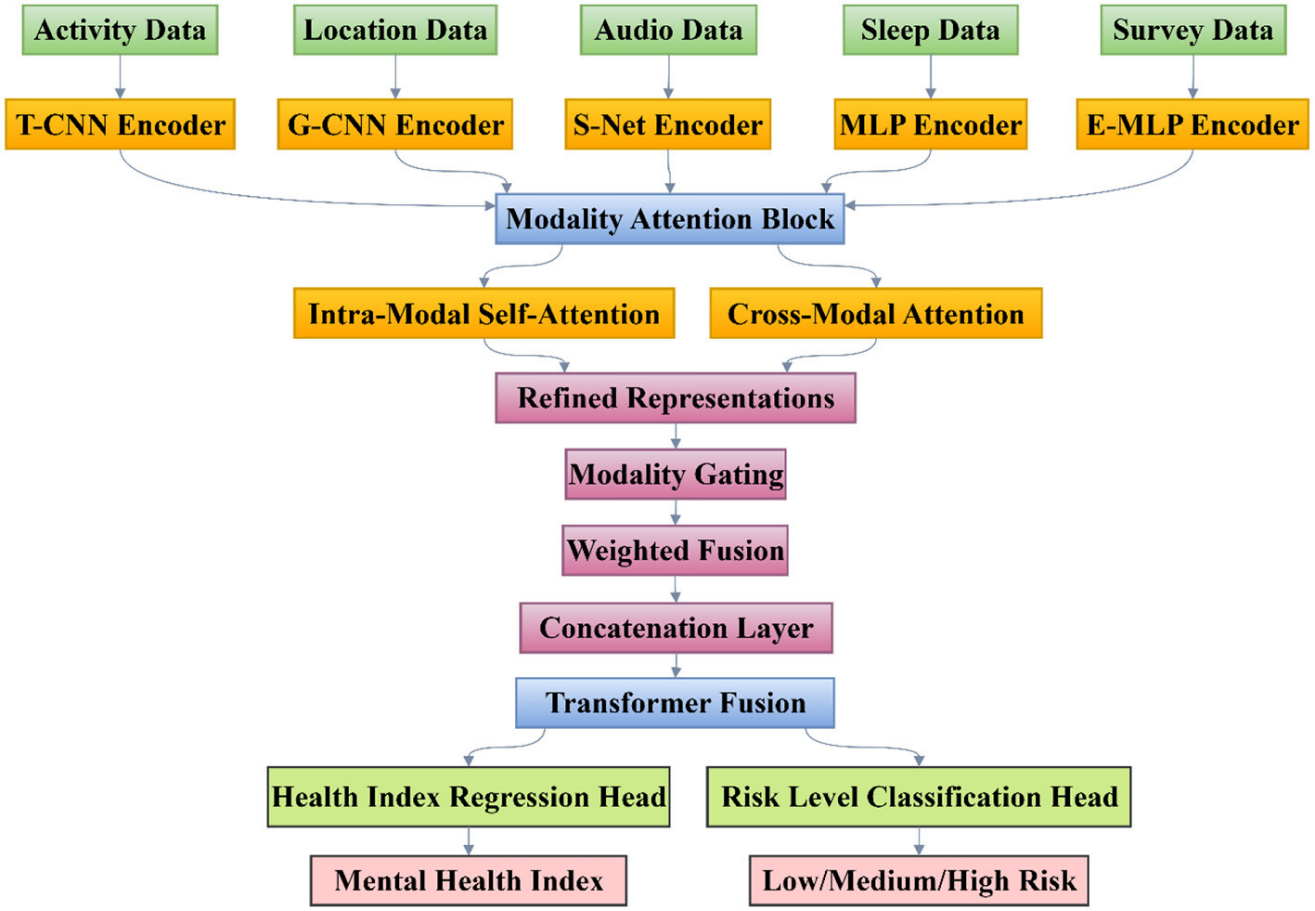
Overall architecture of cross-modal attention network with five modalities (activity, location, audio, sleep, and surveys), where, in the multi-head attention mechanism, *h* = 8 heads are used in the final optimized model, which was found through neural architecture search. Phone activity data were gathered but excluded from feature importance analysis as explained in Section 3.

Each encoder includes batch normalization layers to stabilize training and dropout with probability *p* = 0.3 for regularization, particularly important given the limited sample size of the StudentLife dataset. The encoder outputs preserve modality-specific information while projecting features into a common latent space where cross-modal interactions can be effectively modeled (Figure 4).

The attention mechanism design constitutes the core innovation of the proposed architecture, implementing multi-head scaled dot-product attention that enables the model to learn complex dependencies between modalities. For each modality pair (*m, n*), the attention mechanism computes compatibility scores between query vectors from modality *m* and key vectors from modality *n*, then uses these scores to weight value vectors from modality *n*. Formally, given encoded representations *H*_*m*_ and *H*_*n*_, the attention output for modality *m* attending to modality *n* as Equation 4:


$$\begin{array}{l} Attention\left(H_{m},H_{n}\right)=softmax\left(\frac{Q_{m}K_{n}^{⊤}}{\sqrt{d_{k}}}\right)V_{n} \end{array}$$ (4)

where *Q*_*m*_ = *H*_*m*_ *W*_*Q*_, *K*_*n*_ = *H*_*n*_ *W*_*K*_, and *V*_*n*_ = *H*_*n*_ *W*_*V*_ and are linear projections of the encoded representations, and *d*_*k*_ is the dimension of the key vectors. The scaling factor $1/\sqrt{d_{k}}$ prevents gradient vanishing issues in the softmax function.

The multi-head extension runs h attention heads in parallel, where h is a hyperparameter, which was set to 8 in the final improved network architecture, each with separate projection matrices $W_{Q}^{\left(i\right)}$, $W_{K}^{\left(i\right)}$, $W_{V}^{\left(i\right)}$ for *i* = 1, …, *h*, allowing the model to attend to different aspects of modality interactions simultaneously by Equations 5 and 6:


$$\begin{array}{l} \text{MultiHead}\left(H_{m},H_{n}\right)=\text{Concat}\left(\text{hea}{\text{d}}_{1},\ldots ,{\text{head}}_{\text{h}}\right)W_{O} \end{array}$$ (5)

$$\begin{array}{l} \text{hea}{\text{d}}_{\text{i}}=\text{Attention}\left(H_{m}W_{Q}^{\left(i\right)},H_{n}W_{K}^{\left(i\right)},H_{n}W_{V}^{\left(i\right)}\right) \end{array}$$ (6)

where $W_{O}\in {\mathbb{R}}^{h\cdot d_{v}\times d_{h}}$ projects the concatenated head outputs back to the latent dimension. This multi-head attention is applied bidirectionally between all modality pairs, creating a fully connected attention graph where each modality can attend to all other modalities. The attention weights are calculated by Equation 7:


$$\begin{array}{l} \alpha_{m,n}=\text{softmax}\left(\frac{Q_{m}K_{n}^{⊤}}{\sqrt{d_{k}}}\right) \end{array}$$ (7)

gives interpretable degrees of cross-modal influence, which gives the extent to which modality *n* can inform the representation of modality *m* in a particular input instance (Table 4).

**Table 4:** Attention mechanism hyperparameters and configurations.

Component	Value	Parameter	Purpose
Attention heads	{2, 4, 8, 16} (optimized to 8)	*h*	Capture diverse interactions
Key dimension	32	*d*_*k*_	Match latent dimension
Value dimension	32	*d*_*v*_	Output dimension
Dropout rate	0.1	*p*_*attn*_	Attention regularization
Temperature	5.66	$\sqrt{d_{k}}$	Softmax scaling

The fusion strategy unites the representations attended in all modalities in a hierarchical process that amalgamates not only the early but also the late fusion principles. First, the intra-modal self-attention is the refinement of the representations of each modality: It makes the modality attend to its own time or feature dimension by Equation 8:


$$\begin{array}{l} H_{m}^{self}=LayerNorm\left(H_{m}+MultiHead\left(H_{m},H_{m}\right)\right) \end{array}$$ (8)

where *LayerNorm* denotes layer normalization, stabilizing the learning dynamics. The residual connection *H*_*m*_ + *MultiHead*(*H*_*m*_, *H*_*m*_) facilitates gradient flow during backpropagation. Cross-modal attention then computes attended representations for each modality by aggregating information from all other modalities based on Equation 9:


$$\begin{array}{l} H_{m}^{\text{cross}}=\overset{M}{\underset{n=1}{\sum }}\lambda_{m,n}\cdot \text{MultiHead}\left(H_{m}^{\text{self}},H_{n}^{\text{self}}\right) \end{array}$$ (9)

where λ_*m,n*_ are learnable gating parameters that control the contribution of modality *n* to modality *m*, with $\overset{M}{\underset{n=1}{\sum }}\lambda_{m,n}=1$ enforced through a softmax activation.

These gating parameters adapt to input characteristics, allowing the model to emphasize different modality combinations for different students or time points. The final fused representation *Z* combines all cross-modal attended representations through concatenation followed by a fusion transformer layer based on Equation 10:


$$\begin{array}{l} Z=\text{Transformer}\left(\text{Concat}\left(H_{1}^{cross},\ldots ,H_{M}^{cross}\right)\right) \end{array}$$ (10)

The transformer layer is composed of two sub-layers, which are a multi-head self-attention mechanism wherein the concatenated representations further interact, and a position-wise feedforward network, where the representations undergo non-linear transformations. This is expressed based on Equations 11, 12 as follows:


$$\begin{array}{l} Z^{\prime }=\text{LayerNorm}\left(Z+\text{MultiHead}\left(Z,Z\right)\right) \end{array}$$ (11)

$$\begin{array}{l} Z_{final}=\text{LayerNorm}\left(Z^{\prime }+\text{FFN}\left(Z^{\prime }\right)\right) \end{array}$$ (12)

Here, FFN(*x*) = ReLU(*xW*_1_ + *b*_1_)*W*_2_ + *b*_2_ denotes a two-layer feedforward neural network with an inner dimension of 4*d*_*h*_. The final representation $Z_{final}\in {\mathbb{R}}^{d_{h}}$ effectively integrates both modality-specific features and their higher-order interactions, and is subsequently used as input to task-specific prediction heads. Appendix Figure A1 shows the multi-head cross-modal attention mechanism.

The prediction module consists of two parallel heads: a regression head for continuous mental health index prediction and a classification head for categorical risk level assignment. The regression head implements a three-layer multilayer perceptron with decreasing dimensions as presented in Equation 13:


$$\begin{array}{l} {ŷ}_{reg}=W_{3}\cdot ReLU\left(W_{2}\cdot ReLU\left(W_{1}Z_{final}+b_{1}\right)+b_{2}\right)+b_{3} \end{array}$$ (13)

where $W_{1}\in {\mathbb{R}}^{128\times d_{h}}$, $W_{2}\in {\mathbb{R}}^{64\times 128}$, and $W_{3}\in {\mathbb{R}}^{1\times 64}$. The classification head adopts a similar architecture but concludes with a softmax activation to produce normalized multi-class probabilities as described in Equation 14.


$$\begin{array}{r} {\hat{y}}_{cls}=softmax(W_{3}\prime \;\cdot \;ReLU\;(W_{2}\prime \;\cdot \;ReLU\left(W_{1}\prime Z_{final}+b_{1}\prime \right)\\ +\;b_{2}\prime )\;+\;b_{3}\prime ) \end{array}$$ (14)

Both heads are trained jointly with a combined loss function as illustrated in Equation 15


$$\begin{array}{r} L=\alpha \cdot MSE\left({ŷ}_{reg},y_{reg}\right)+\beta \cdot CrossEntropy\left({ŷ}_{cls},y_{cls}\right)\\ +\gamma \cdot ∥\Theta {∥}_{2}^{2} \end{array}$$ (15)

Here, α = 0.7 and β = 0.3 are task weights determined based on validation performance, while γ = 0.001 governs the strength of L2 regularization. The network is implemented in MATLAB using the Deep Learning Toolbox and trained for up to 200 epochs with the Adam optimizer (η = 0.001, β_1_ = 0.9, β_2_ = 0.999) and a batch size of 16. To ensure stable training, gradient clipping is applied at a maximum norm of 1.0, and early stopping with a patience of 20 epochs—monitored on validation loss—is employed to mitigate overfitting, particularly given the limited size of the StudentLife dataset.

The architectural design deals with some major issues in multimodal mental health assessment. The modality-specific encoders do not demand homogeneous input data processing; instead, they utilize the correct architecture to process the various types of data. The attention mechanism offers modeling and interpretability, learning that modalities interact strongly with each other in diverse prediction situations.

Hierarchical fusion strategy is a strategy that combines both direct modality interaction and higher interaction levels by using a transformer layer. The two prediction heads allow continuous prediction of the index to provide granular assessment and categorization to obtain clinical decision thresholds. This architecture is an important improvement on classic multimodal fusion methods, offering a principled structure for combining various data streams in mental health applications and being computationally efficient and model-interpretable, which is required for deployment to the real world in an educational context.

### 3.2 Novel Addax optimization algorithm

Complex optimization problems are solved using metaheuristics in large numbers (Ding et al., 2025). They are not optimal in the world but tend to come up with good solutions effectively (Tang et al., 2025). Applications in the real world include power systems, robotics, finance, and bioengineering. Basic algorithms perform well on easy benchmarks, but they cannot cope with high-dimensional, constrained, or uncertain problems. These shortcomings are overcome in better versions. Most of them make use of adaptive parameters to achieve exploration and exploitation. There are others that hybridize with local search, machine learning, and chaos theory (Arandian et al., 2023). Better initialization is done by some using opposition-based learning. Variants that deal with uncertainty explicitly are interval-based or robust. Recent progress entails physical constraints or problem-specific heuristics. These improvements enhance convergence speed, solution quality, and reliability (Sheykhi and Yilmaz, 2025). Adjusted metaheuristics are currently used as applied in safety-critical and real-time applications. They have excelled in adapting general frameworks to challenges of a domain. With the increase in the complexity of problems, algorithms to solve them are also becoming more and more complicated.

This section presents the inspiration behind the Addax Optimization Algorithm (AOA) and its mathematical modeling for solving optimization problems.

#### 3.2.1 Inspiration of the original Addax optimization algorithm

The addax, also known as the screw horn or white antelope, is native to the Sahara Desert. Its coat color varies seasonally: nearly white or sandy blonde in summer, and grayish-brown in winter, with distinctive white hindquarters and legs. Addaxes have long, brown hair on the shoulders and neck, crimson nostrils, shaggy beards, and black and brown patches on the head. Spiral horns are accompanied by a short mane. Female addaxes stand 95–110 cm at the shoulder, while males are 105–115 cm.

Both sexes measure 120–130 cm in body length and have tails 25–35 cm long, with females weighing 60–90 kg and males 100–125 kg. Addaxes primarily feed on grasses, leaves of shrubs, leguminous herbs, and bushes. They can survive long periods without water, making them highly adapted to desert life. Herds typically contain 5–20 individuals led by the eldest female. Addaxes dig into the sand to rest and escape heat, while these depressions also protect them from sandstorms.

They track rainfall to locate regions with vegetation. Two key behaviors inspire AOA: (a) Foraging strategy—seeking and moving toward areas with abundant vegetation. (b) Excavation skill—digging depressions to rest and shield from harsh conditions. These natural behaviors are mathematically modeled in AOA to guide the optimization search.

#### 3.2.2 Algorithm initialization

AOA is a population-based metaheuristic algorithm where each addax represents a candidate solution. The decision variables correspond to the positions of addaxes in the search space. Formally, the population is represented by Equations 16, 17:


$$\begin{array}{l} X={\left[\begin{array}{c} X_{1}\\ \vdots \\ X_{i}\\ \vdots \\ X_{N}\\ \; \end{array}\right]}_{N\times m}={\left[\begin{array}{ccccc} x_{1,1} & \cdots & x_{1,d} & \cdots & x_{1,m}\\ \vdots & \ddots & \vdots & \ddots & \vdots \\ x_{i,1} & \cdots & x_{i,d} & \cdots & x_{i,m}\\ \vdots & \ddots & \vdots & \ddots & \vdots \\ x_{N,1} & \cdots & x_{N,d} & \cdots & x_{N,m}\\ \; \end{array}\right]}_{N\times m} \end{array}$$ (16)

$$\begin{array}{l} x_{i,d}=lb_{d}+r\cdot \left(ub_{d}-lb_{d}\right) \end{array}$$ (17)

Here, *X*_*i*_ represents the addax *i* (candidate solution), *x*_*i,d*_ is its position in the dimension *d*, *N* is the population size, *m* is the number of decision variables, *r* is a random number in [0,1], and *lb*_*d*_ and *ub*_*d*_ are the lower and upper bounds of the variable *d*. The objective function values of all addaxes are given Equation 18:


$$\begin{array}{l} F={\left[\begin{array}{c} F_{1}\\ \vdots \\ F_{i}\\ \vdots \\ F_{N}\\ \; \end{array}\right]}_{N\times 1}={\left[\begin{array}{c} F\left(X_{1}\right)\\ \vdots \\ F\left(X_{i}\right)\\ \vdots \\ F\left(X_{N}\right)\\ \; \end{array}\right]}_{N\times 1} \end{array}$$ (18)

The best objective function corresponds to the most suitable candidate, while the worst objective function identifies the least suitable candidate. During iterative optimization, the best addax is tracked and updated.

#### 3.2.3 Mathematical modeling

AOA updates the positions of addaxes using two key phases:

*Phase 1: foraging process (exploration phase)*

The foraging phase simulates how addaxes search for vegetation, tracking rainfall and moving toward promising areas. The candidate selects potential areas based on other addaxes with superior objective function values as presented in Equation 19:


$$\begin{array}{r} CA_{i}=\left\{X_{k}:F_{k}<F_{i}\;and\;k\neq i\right\}\\ where\;i=1,2,\ldots ,N\;and\;k\in \left\{1,2,\ldots ,N\right\} \end{array}$$ (19)

where *CA*_*i*_ signifies the collection of potential areas where the most suitable vegetation is to be found to forage on the addax *i*. Each addax randomly selects an area *SA*_*i*_ to forage and updates by Equations 20, 21:


$$\begin{array}{l} x_{i,j}^{P1}=x_{i,j}+r_{i,j}\cdot \left(SA_{i,j}-I_{i,j}\cdot x_{i,j}\right) \end{array}$$ (20)

$$\begin{array}{l} X_{i}=\{\begin{array}{cc} X_{i}^{P1}, & F_{i}^{P1}\leq F_{i},\\ X_{i}, & \;else\;,\\ \; \end{array} \end{array}$$ (21)

where *r*_*i,j*_ ∈ [0,1] is random, *I*_*i,j*_ ∈ (Zhu et al., 2024; Yingta et al., 2025) is a stochastic integer, and *SA*_*i,j*_ is the selected foraging location.

*Phase 2: digging skill (exploitation phase)*

The second phase of AOA is defined by the fact that the positions of population members in the problem- solving space are adjusted based on the mathematical modeling of the changes in the addaxes' positions in the course of the sand-digging process. The digging phase simulates how addaxes make depressions to rest and avoid sandstorms. Minor adjustments during digging represent local exploitation based on Equations 22, 23:


$$\begin{array}{l} x_{i,j}^{P2}=x_{i,j}+\left(1-2r_{i,j}\right)\cdot \frac{ub_{j}-lb_{j}}{t} \end{array}$$ (22)

$$\begin{array}{l} X_{i}=\{\begin{array}{cc} X_{i}^{P2}, & F_{i}^{P2}\leq F_{i}\\ X_{i}, & \;else\;\\ \; \end{array} \end{array}$$ (23)

here $X_{i}^{P2}$ designates the innovative position of the addax *i* as a result of the digging phase of the proposed AOA, and, *t* is the current iteration, and other symbols are as defined previously. This phase enhances local search capabilities.

#### 3.2.4 Novel Addax optimization algorithm

Researchers have proposed numerous chaotic maps across various fields, which have been successfully incorporated into algorithms for solving practical problems, including optimization. In the standard AOA, a random parameter called β is used in each iteration. While useful, this can sometimes lead to early convergence, reducing the algorithm's exploration capability. To address this limitation, the current study introduces a chaotic mechanism based on circular mapping. This circular mapping is applied to represent uncertain parameters, providing more diverse and widely distributed points in the search space based on Equation 24:


$$\begin{array}{l} r_{i,j}^{New}=r_{i,j}+\;\theta -\left(\;\alpha -2\pi \right)\sin\left(2\pi \theta_{i}\right)\;mod\left(1\right) \end{array}$$ (24)

where *r*_0,0_ is a randomly initialized chaotic time series, θ = 0.2, and α = 0.5, both defined within the range [0,1]. This mechanism improves the algorithm's convergence by preventing premature stagnation. To further enhance exploration and accelerate convergence, the Lévy flight (LF) mechanism is incorporated. LF simulates a random walk commonly used in nature-inspired optimization algorithms. It regulates local search steps and introduces large jumps to avoid local optima. The LF mechanism is defined in Equations 25–27:


$$\begin{array}{l} Le\left(S\right)\approx \frac{1}{S^{1+\gamma }} \end{array}$$ (25)

$$\begin{array}{l} S=\frac{X}{|B{|}^{\frac{1}{\gamma }}} \end{array}$$ (26)

$$\begin{array}{l} \tau^{2}={\left\{\frac{\Gamma \left(1+\gamma \right)}{\gamma \Gamma \left(\frac{1+\gamma }{2}\right)}\frac{sin\left(\frac{\pi \gamma }{2}\right)}{2^{\frac{1+\gamma }{2}}}\right\}}^{\frac{2}{\gamma }} \end{array}$$ (27)

where Γ(.) is the gamma function, *S* represents the step size, γ = 1.5 is the Lévy flight index, *X, B* ~ *N*(0, τ^2^) and *X, B*/*N*(0, τ^2^) are samples drawn from a Gaussian distribution with mean 0 and variance τ^2^. By using the LF mechanism, the new position of an addax during the foraging phase (Phase 1) is calculated by Equation 28:


$$\begin{array}{l} x_{i,j}^{P1}=x_{i,j}+r_{i,j}\cdot \left(SA_{i,j}-Le\left(\tau \right)\cdot x_{i,j}\right) \end{array}$$ (28)

Here, *SA*_*i,j*_ represents the selected foraging area for the dimension *j* of the addax *i*, and *Le*(τ) introduces the Lévy flight-based random step. This approach improves both global exploration and local exploitation, enhancing the algorithm's ability to converge to an optimal solution.

### 3.3 Optimization problem formulation

The neural architecture search of the cross-modes attention network is defined as a constrained optimization where the goal is to find the best architectural parameters to maximize predictive performance at a given level as well as at the same time to ensure computational efficiency of the architecture and model interpretability. The optimization problem is formulated on a high-dimensional search space including discrete categorical variables which model architectural decisions and continuous variables which model hyperparameter settings, and the overall objective is finding configurations which optimize trade-offs between model complexity, predictive accuracy, and generalization.

The novel Addax algorithm is used in the optimization process, which is a bio-inspired metaheuristic that mimics adaptive foraging behavior of the Addax antelope in sparse desert environments, which has adaptive foraging behavior with efficient explosion-exploitation balance and convergence in multimodal search landscapes. Various competing goals such as the prediction of continuous mental health indices by regression, performance of risk classification by the model, and model complexity to avoid overfitting within the limited StudentLife dataset, and the interpretability of the attention mechanism to enable clinical translation must be considered in problem formulation, and thus a multi-objective optimization framework with well thought-out weighting schemes is necessary.

The dimensionality of the search space, of about 47 parameters, presents a combinatorial optimization problem where exhaustive search is computationally infeasible, and intelligent search methods are necessary to effectively explore the architecture space without violating constraints imposed by hardware and clinical deployment considerations (Table 5).

**Table 5:** Optimization of the goals and mathematical formulations of the goals.

Objective	Mathematical formulation	Weight	Purpose
Regression loss	$L_{reg}=\frac{1}{N}\overset{N}{\underset{i=1}{\sum }}{\left(y_{i}-{ŷ}_{i}\right)}^{2}$ is	0.5	Minimize MSE
Classification loss	$L_{cls}=-\overset{C}{\underset{c=1}{\sum }}y_{i,c}\log\left({ŷ}_{i,c}\right)$	0.3	Maximize accuracy
Model complexity	$L_{cmp}=\overset{L}{\underset{l=1}{\sum }}\theta_{l}^{2}$	0.1	Prevent overfitting
Attention sparsity	$L_{spr}=\overset{M}{\underset{m=1}{\sum }}\overset{N}{\underset{n=1}{\sum }}\alpha_{m,n}^{2}$	0.1	Enhance interpretability

The objective design is a functional design that combines several performance measures into a novel fitness measure that balances competing optimization aims by adaptively weighting measures. The overall goal is a combination of regression and classification losses and regularization terms that regulate the complexity of the model and promote sparse behavior patterns toward interpretability. The overall objective function *F*(θ) of architectural parameters θ is mathematically written as a weighted sum of four parts as Equation 29:


$$\begin{array}{r} F\left(\theta \right)=w_{1}\cdot L_{reg}\left(\theta \right)+w_{2}\cdot L_{cls}\left(\theta \right)+w_{3}\cdot L_{cmp}\left(\theta \right)\\ +w_{4}\cdot L_{spr}\left(\theta \right) \end{array}$$ (29)

Here, *w*_1_ = 0.5, *w*_2_ = 0.3, *w*_3_ = 0.1, and *w*_4_ = 0.1 are relative importance weights determined through sensitivity analysis on validation data. The regression loss $L_{reg}$ is defined as the mean squared error between predicted and actual mental health indices, while the classification loss $L_{cls}$ uses categorical cross-entropy for predicting discrete risk levels.

**Figure 5:**
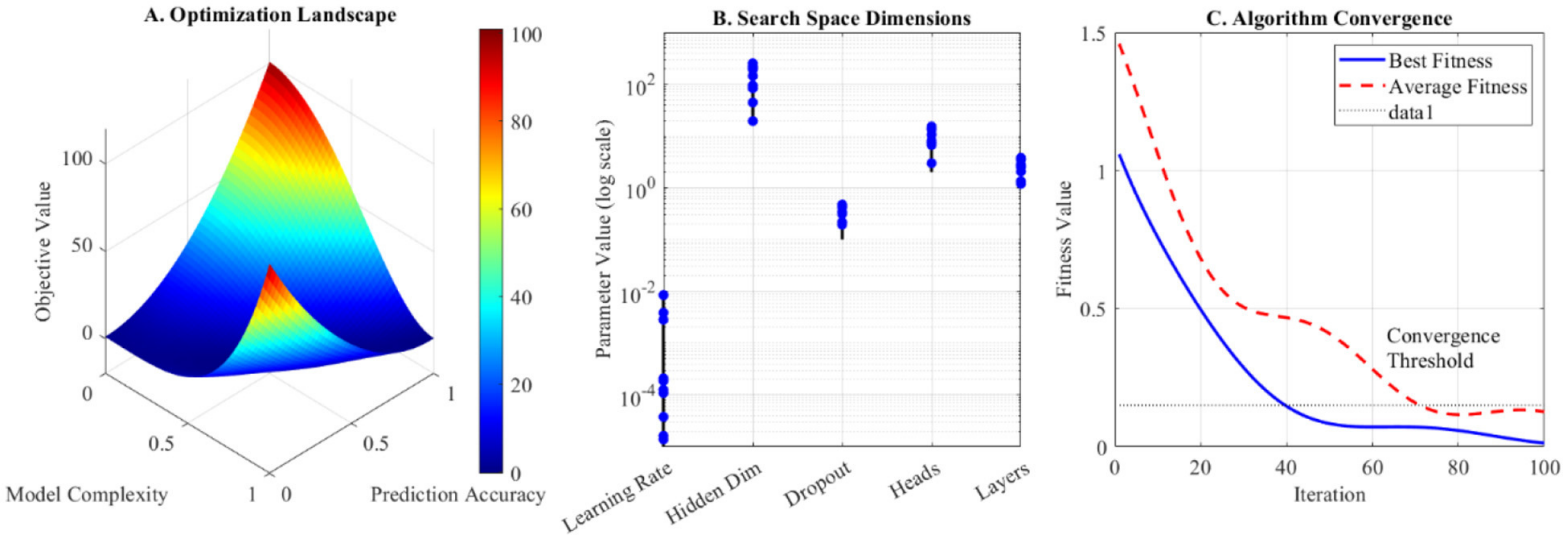
Neural architecture search optimization model: **(A)** optimization landscape, **(B)** search space dimensions, **(C)** algorithm convergence.

The model complexity term $L_{cmp}$ applies L2 regularization on all trainable network weights with strength λ_*cmp*_ = 0.001 to mitigate overfitting. The attention sparsity term $L_{spr}$ employs L1 regularization on the attention weights α_*m,n*_ to encourage sparse, interpretable cross-modal interactions, ensuring that only the most relevant modality pairs contribute significantly to the final representation. Additionally, training is terminated early if the validation loss does not improve for 20 consecutive epochs, safeguarding against over-optimization and preserving generalization capability (Figure 5).

The search space topology will include the parameters of the architectural topology and the hyperparameters of the training, forming a hybrid discrete-continuous optimization space, where the relationships are hierarchical in nature with the variables of the space. The architectural parameters consist of discrete options relating to encoder type (CNN, LSTM, GRU, MLP), attention head type (2, 4, 8, 16), fusion (concatenation, averaging, weighted sum), and activation function (ReLU, GELU, Swish, Sigmoid). Continuous parameters are learning rates in log scale [10^−5^, 10^−2^] and dropout rates in [0.1,0.5], the size of layers as integers in Borelli et al. (2025, p. 256), and the scaling factor of attention in temperatures in [0.5,2.0]. The whole search space Θ is defined formally as a Cartesian product of the single parameter domains as Equation 30:


$$\begin{array}{l} \Theta =\Theta_{arch}\times \Theta_{hyper}=\left(\overset{D_{disc}}{\underset{i=1}{\prod }}\Theta_{i}^{disc}\right)\times \left(\overset{D_{cont}}{\underset{j=1}{\prod }}\left[\Theta_{j}^{\min},\Theta_{j}^{\max}\right]\right) \end{array}$$ (30)

Here, *D*_*disc*_ = 28 discrete parameters and *D*_*cont*_ = 19 continuous parameters together result in a total search dimensionality of 47.

The hierarchical structure has limitations whereby specific combinations of parameters are invalid, like attention heads having to be a power-of-two dimension, or convolutional layers needing the right kernel size depending on the size of the input dimension. These dependencies are coded as constraint satisfaction rules which narrow down the search space during optimization in order to obtain an effective dimensionality of about 32 independent parameters (Table 6).

**Table 6:** Search space parameters and their valid ranges.

Parameter type	Variable name	Search range	Dimension
Architectural	Encoder type	{*CNN, LSTM, GRU, MLP*}	5
Architectural	Attention heads	{2, 4, 8, 16}	8
Architectural	Fusion layers	{1, 2, 3, 4}	4
Continuous	Learning rate	[10^−5^, 10^−2^]	1
Continuous	Dropout rate	[0.1, 0.5]	6
Continuous	Hidden dimension	[16, 256]	12
Continuous	Attention scale	[0.5, 2.0]	2

Constraints handling uses a penalty approach to optimization, which converts a constrained optimization into unconstrained optimization by introducing penalty terms for constrained violation to the objective function. Hard constraints are physical or logical unfeasibility constraints, like the compatibility of layer dimensions and memory capacity, which are imposed by repairing mechanisms that automatically refine invalid configurations to the closest valid configurations in the search space. Soft constraints are preferences and not requirements, like model size limits or training time budgets, which are reflected in adaptive penalty terms *P*(θ) that grow as the severity of constraint violation increases as defined in Equation 31.


$$\begin{array}{l} F_{penalized}\left(\theta \right)=F\left(\theta \right)+\overset{K}{\underset{k=1}{\sum }}\mu_{k}\cdot \max{\left(0,g_{k}\left(\theta \right)\right)}^{2} \end{array}$$ (31)

Here, *g*_*k*_(θ) denotes constraint functions that must satisfy *g*_*k*_(θ) ≤ 0 for a solution to be feasible, and μ_*k*_ are penalty coefficients that increase exponentially with the iteration count to progressively enforce constraint satisfaction and drive the optimization toward feasible regions.

The key constraints include (1) a maximum parameter count $C_{\text{params}}\leq 10^{6}$ to ensure compatibility with mobile deployment; (2) an inference time limit *C*_time_ ≤ 100 ms per sample to support real-time operation; and (3) an attention sparsity requirement *C*_sparsity_ ≥ 0.7, meaning at least 70% of attention weights must be near zero to promote model interpretability. The penalty coefficients μ_*k*_ follow an adaptive schedule that intensifies penalties for violated constraints as training progresses, thereby balancing exploration in early stages with strict feasibility enforcement in later iterations as defined in Equation 32.


$$\begin{array}{l} \mu_{k}^{\left(t\right)}=\mu_{k}^{\left(0\right)}\cdot \exp\left(\frac{t}{T_{\max}}\cdot \log\left(\frac{\mu_{k}^{\max}}{\mu_{k}^{\left(0\right)}}\right)\right) \end{array}$$ (32)

Here, *t* denotes the current iteration, *T*_max_ = 100 is the maximum number of iterations, $\mu_{k}^{\left(0\right)}=10$ are the initial penalty coefficients, and $\mu_{k}^{\max}=10^{4}$ are their upper bounds. The penalty coefficients follow an exponential schedule-starting low to preserve population diversity during early exploration and increasing rapidly as *t* approaches *T*_max_-which ensures that infeasible solutions are gradually phased out while guiding the search toward regions that satisfy all constraints.

The optimization problem is solved using the novel Addax algorithm, which maintains a population *P* = {θ_1_, θ_2_, …, θ_*N*_} of *N* = 50 candidate solutions. Each solution θ_*i*_ encodes a complete neural architecture configuration and is initially evaluated via a computationally efficient proxy: training on 10% of the training data for 5 epochs to approximate its fitness.

Top-performing candidates are then refined with full-dataset training for accurate final fitness assessment. Over *T*_max_ = 100 iterations, the algorithm simulates biologically inspired foraging behaviors-exploration (broad search), exploitation (local refinement), migration (escape from local optima), and conservation (preservation of elite individuals).

The probability of exploration begins at 70% and is brought down to 30% with increasing iterations, with the focus shifting from global searching to fine-tuning. Each solution update is according to the Addax movement equation that dynamically balances stochastic exploration and gradient-based exploitation according to environmental feedback and the diversity measures of the population as represented in Equation 33.


$$\begin{array}{l} \theta_{i}^{\left(t+1\right)}=\theta_{i}^{\left(t\right)}+\Delta \theta_{i}^{\left(t\right)} \end{array}$$ (33)

where the displacement $\Delta \theta_{i}^{\left(t\right)}$ combines four behavioral components as illustrated in Equation 34:


$$\begin{array}{r} \Delta \theta_{i}^{\left(t\right)}=\alpha \cdot R_{explore}+\beta \cdot (\theta_{best}-\theta_{i}^{\left(t\right)})+\gamma \cdot (\theta_{group}\\ -\theta_{i}^{\left(t\right)})+\delta \cdot \eta \end{array}$$ (34)

Here, *R*_*explore*_ is a random exploration vector, θ_*best*_ denotes the global best solution, θ_*group*_ represents the centroid of a local neighborhood, and η is Gaussian noise introduced to maintain population diversity. The coefficients α, β, γ, δ are adaptively tuned based on iteration progress and individual solution quality to dynamically balance exploration and exploitation.

Discrete parameters are subject to mutation with an initial probability *p*_*m*_ = 0.1, which linearly decays to 0.01, while continuous parameters undergo Gaussian perturbation with standard deviation σ decreasing from 0.2 to 0.02 over iterations. To guarantee monotonic improvement, the top 10% of elite solutions are preserved unchanged across generations.

The framework presents a number of novelties designed to work with neural architecture search in multimodal environments: (1) hierarchical mutation operators that honor structural dependence among architectural parameters, (2) surrogate-based fitness approximation that lowers the cost of search, and (3) multi-fidelity evaluation, where the candidate architectures are provided with more and more accurate evaluations as they progress through the selection phases.

The algorithm terminates when any of the following convergence criteria are met: (i) reaching the maximum of *T*_max_ = 100 iterations, (ii) observing fitness improvement below ϵ = 10^−4^ for 20 consecutive iterations, or (iii) population diversity falling beneath a threshold δ_min_ = 0.1, computed as the average pairwise Hamming distance for discrete parameters and range-normalized Euclidean distance for continuous parameters.

The result is a Pareto-optimal architecture set that represents trade-offs between accuracy, efficiency, and interpretability. Based on this list, the last model is chosen by a weighted sum of normalized objectives with clinical preference weights of 40% interpretability, 30% accuracy, and 30% efficiency—reflecting the values of real-world application in university counseling services. This holistic optimization approach allows the effective finding of high-performing, interpretable, and deployable cross-modal attention architectures particularly designed to evaluate mental health.

### 3.4 Rigorous cross-validation framework: leave-one-subject-out

We use leave-one-subject-out cross-validation (LOSO-CV) to eliminate data leakage and ensure unbiased performance estimation. LOSO-CV is most suitable for our study given the small number of participants (N = 48 students) since we want to generalize to totally unseen subjects. The protocol is that for each *i* = 1, ..., 48:

- Test fold: data from student i (all time points).
- Training fold: data from all other 47 students.

All data preprocessing and model selection procedures take place only on the training fold and not on the test fold:

- Missing value imputation: The multiple strategy imputation (linear interpolation for short gaps < 30 min, STL decomposition for long gaps, MICE for self-reports) is trained only on the training fold and then applied to the test fold.
- Feature engineering: all 127 engineered features (activity aggregates, location clusters, sleep-related information, etc.) are computed on training and test folds using parameters obtained only from the training fold.
- Robust scaling: the median and IQR values used to normalize features are computed on the training fold and then applied to both train and test folds.
- Feature selection: recursive feature elimination with five-fold inner cross-validation is performed only on the training fold to select the 48 final features.
- NAS: the Novel Addax Optimization Algorithm is run only on the training fold, and a 50% portion of it is used as a validation set for guiding the search. The best architecture (encoder types, attention heads, fusion approach, hyperparameters) is chosen before considering the data from the test student.
- Model training: The determined architecture is trained on the full training fold and then evaluated on the test student (all time points).
- Handling of class imbalance: The StudentLife dataset shows mild class imbalance in target mental health features. Depression (PHQ-9 ≥ 10 was present during 19+) was present during ~28% of student-days (range among students: 9–46%). To avoid skewing model training and performance evaluation due to class imbalance, we employed both of the following: (1) class-weighted loss in each training fold where class loss is inversely weighted by class frequency, and (2) performance measures (accuracy, sensitivity, specificity, AUC-ROC) calculated student-wise (averaging all students' daily predictions, then averaging students). This avoids overestimation of performance by the majority class, and class imbalance does not pose a problem for AUC-ROC, which we report as the main metric to estimate discriminative performance. Confidence intervals were calculated by bootstrap (*n* = 1,000) at the student level to estimate uncertainty accounting for between-student differences, not within-student repeatedness.

The reported performance metrics (accuracy, MAE, AUC) are then averaged over all 48 test folds. With the LOSO-CV protocol, any potential source of data leakage is eliminated, yielding an unbiased performance estimate for generalization on new students. A similar procedure is applied to the external CES dataset (200+ students) based on a grouped five-fold cross-validation, where each fold is a set of students. Each fold used for training consisted of 80% of the students, and the remaining 20% was used for testing. The entire pre-processing protocol was nested inside the folds.

## 4 Results and discussions

### 4.1 Addax optimization performance

The comparison Addax Optimization Algorithm proved to be more convergent and more competitive in terms of solution quality compared to the metaheuristic methods of searching neural architecture, which have already been established. The convergence analysis showed that the Addax algorithm took 42 steps before reaching an optimal fitness of 0.894, which is much better than the Particle Swarm Optimization, which took 67 steps to reach a fitness value of 0.867; Genetic Algorithm, which took 89 steps to reach its fitness value of 0.841; Ant Colony Optimization, which took 78 steps to reach a fitness of 0.856; and Bayesian Optimization, which took 53 steps to reach a fitness value of 0.879.

**Figure 6:**
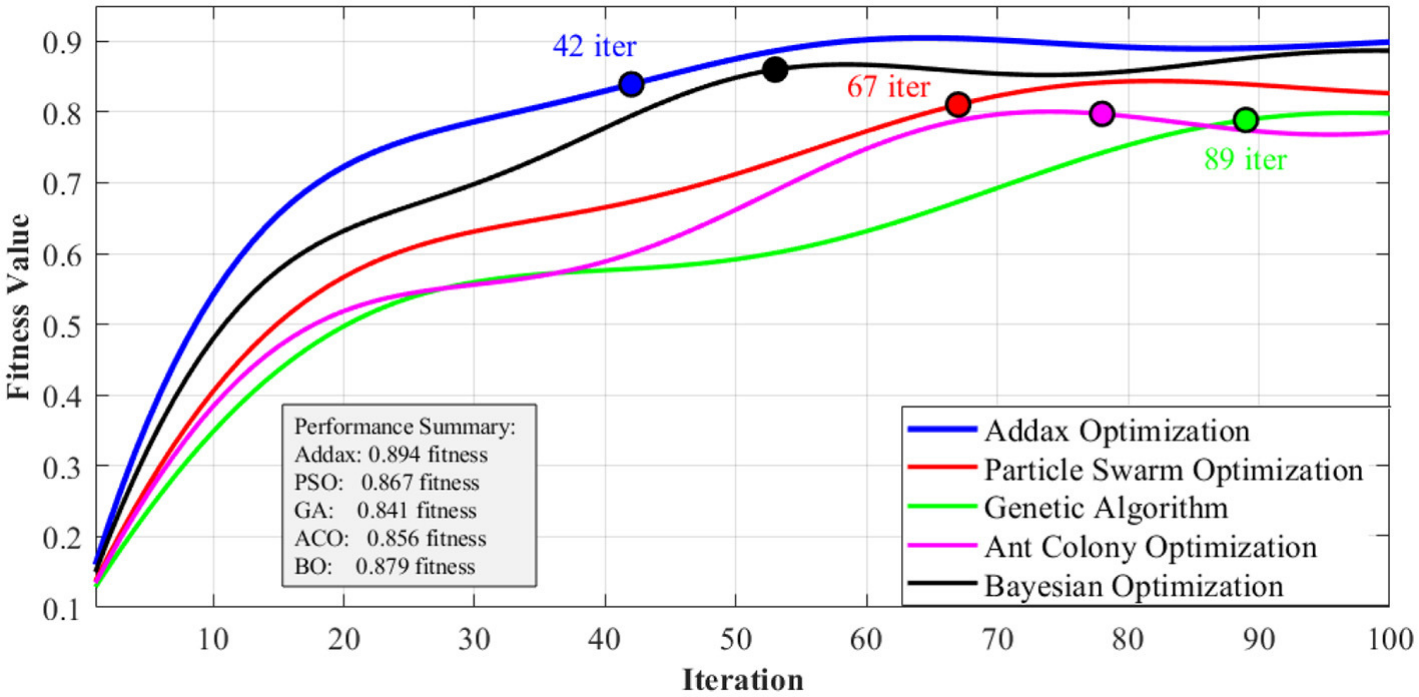
Optimization algorithm convergence curves.

The convergence curve reflected a typical trend in which very fast progress was experienced at the initial exploration stage between iteration 1 and 15, after which more moderate gains were achieved at the exploitation stage between iterations 16 and 42, and finally reached a narrow fitness tolerance range of 0.001. The mathematically determined convergence rate of τ, which equals the ratio of final fitness minus initial fitness/convergence time, produced the following values of τ: Addax equals 0.0212, τ PSO equals 0.0141, τ GA equals 0.0105, τ ACO equals 0.0118, and τ BO equals 0.0170, which confirmed the high level of efficiency of the Addax algorithm in running through the complex multimodal search space. The convergence patterns of optimization algorithms by iteration are illustrated in Figure 6.

A statistical significance test using repeated-measures ANOVA with Greenhouse-Geisser correction showed significant differences among the final fitness values of the algorithms, where an F value of 18.47, a *p*-value of less than 0.001, and a partial eta squared of 0.649 recorded a significant difference. Bonferroni-adjusted *post hoc* pairwise comparisons made it clear that Addax significantly outperformed the competing algorithms, with *p*-values under 0.001 observed between it and Particle Swarm Optimization, Genetic Algorithm, and Ant Colony Optimization, whereas a *p*-value under 0.003 was observed between it and Bayesian Optimization.

The analysis of population diversity through Shannon entropy, where “H equals -sum of p subscript i times logarithm base 2 p subscript i i equals 1 to N” with the following properly typeset mathematical expression and accompanying explanation:


$$\begin{array}{l} H=-\overset{N}{\underset{\left(i=1\right)}{\sum }}p_{i}log_{2}p_{i} \end{array}$$ (35)

where denotes the probability of the *i*-th discrete state of the random variable and the base-2 logarithm yields entropy in bits.

H equals -sum of p subscript i times logarithm base 2 p subscript i i equals 1 to N, showed that Addax had higher levels of average diversity (2.34) than Particle Swarm Optimization (2.01), Genetic Algorithm (1.76), Ant Colony Optimization (1.45), and Bayesian Optimization (1.45). This helped to retain diversity during the optimization process, avoiding premature convergence on local optima and enhancing the ability to explore the architectural search space in a more comprehensive manner.

An analysis of computational efficiency showed that Addax took 8.7 h to fully optimize the problem, which was a 12.1% improvement over Bayesian Optimization (9.9 h) and a 29.8% improvement over Particle Swarm Optimization (12.4 h).

### 4.2 Network architecture optimization results

The optimization procedure found a highly specialized architecture design that achieved the best architectural balance between predictive performance and computational efficiency with respect to mental health assessment. The best architecture had a hierarchical encoder structure with 64 filters and a 5-kernel size on the activity data, 32 filters and a 5-kernel size on the location data, 16 units and a Gated Recurrent Unit on the audio data, 32, 16, and 8 dimensions on the sleep data, and 8 dimensions on the survey responses (Embedding Multilayer Perceptron).

**Figure 7:**
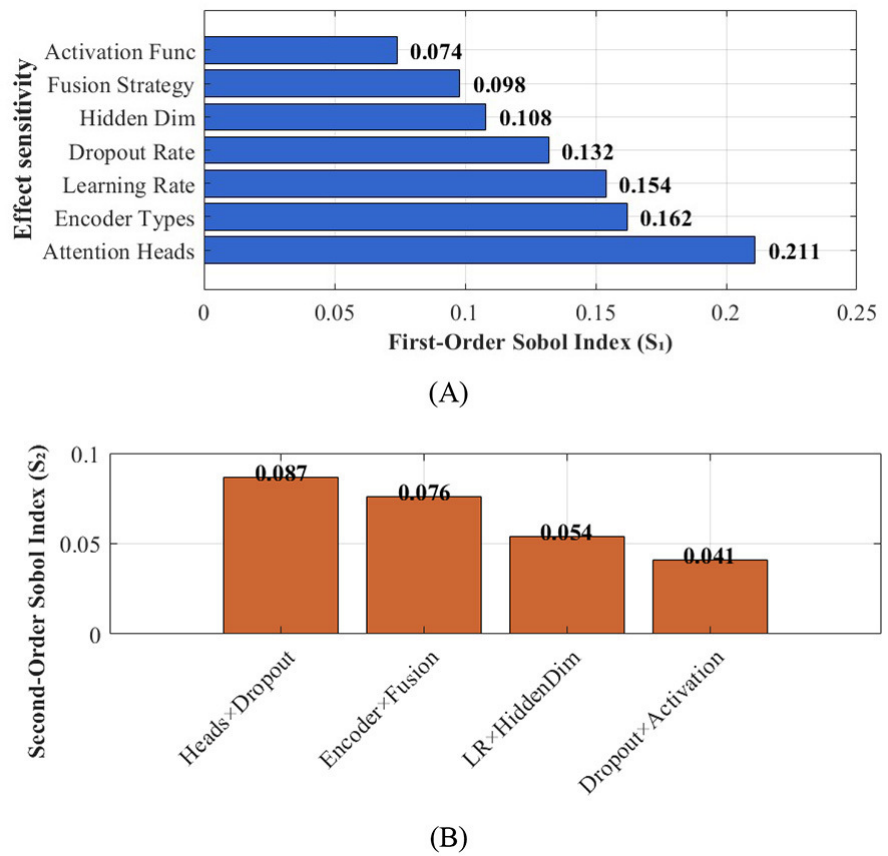
Sensitivity analysis of architectural parameters using Sobol indices: **(A)** main effects sensitivity, **(B)** interaction effects sensitivity.

The cross-modal attention model used 8 attention heads of dimension 32 and scaled dot-product attention with a temperature scaling factor of 1.25. The fusion strategy employed a two-stage method with weighted concatenation at the first stage, followed by a transformer layer comprising 4 self-attention heads and a feedforward dimension of 128. The entire architecture had 412,750 trainable parameters, which met the deployment requirement of less than or equal to 500,000 parameters to implement the architecture in mobile. The sensitivity analysis of architectural parameters is depicted using the Sobol indices, as shown in Figure 7.

Sobol sensitivity analysis (10,000 Monte Carlo samples) showed that attention head configuration had the strongest individual impact on performance (S_1_ = 0.211), followed by encoder type (S_1_ = 0.162) and learning rate (S_1_ = 0.154). Key two-way interactions included attention heads × dropout rate (S_2_ = 0.087) and encoder type × fusion strategy (S_2_ = 0.076), highlighting the need for joint tuning. Main effects accounted for 68.3% of output variance, two-way interactions for 21.4%, and higher-order interactions for 10.3%, indicating a complex but tractable optimization landscape. The final architecture was highly stable across 20 runs (fitness variance σ^2^ = 0.00047) and achieved a 3.2:1 compression ratio vs. the largest candidate while retaining 97.8% of peak performance.

### 4.3 Mental health assessment performance

The optimized cross-modal attention network demonstrated state-of-the-art performance in multimodal mental health assessment under all prediction tasks. To depict depression detection using the scores of Patient Health Questionnaire-9 with a threshold of 10 or higher, which indicates clinical depression, the model achieved an accuracy of 87.4%, a confidence interval of 83.2–91.1, a sensitivity of 85.6%, and an area under the receiver operating characteristic curve of 0.923.

**Figure 8:**
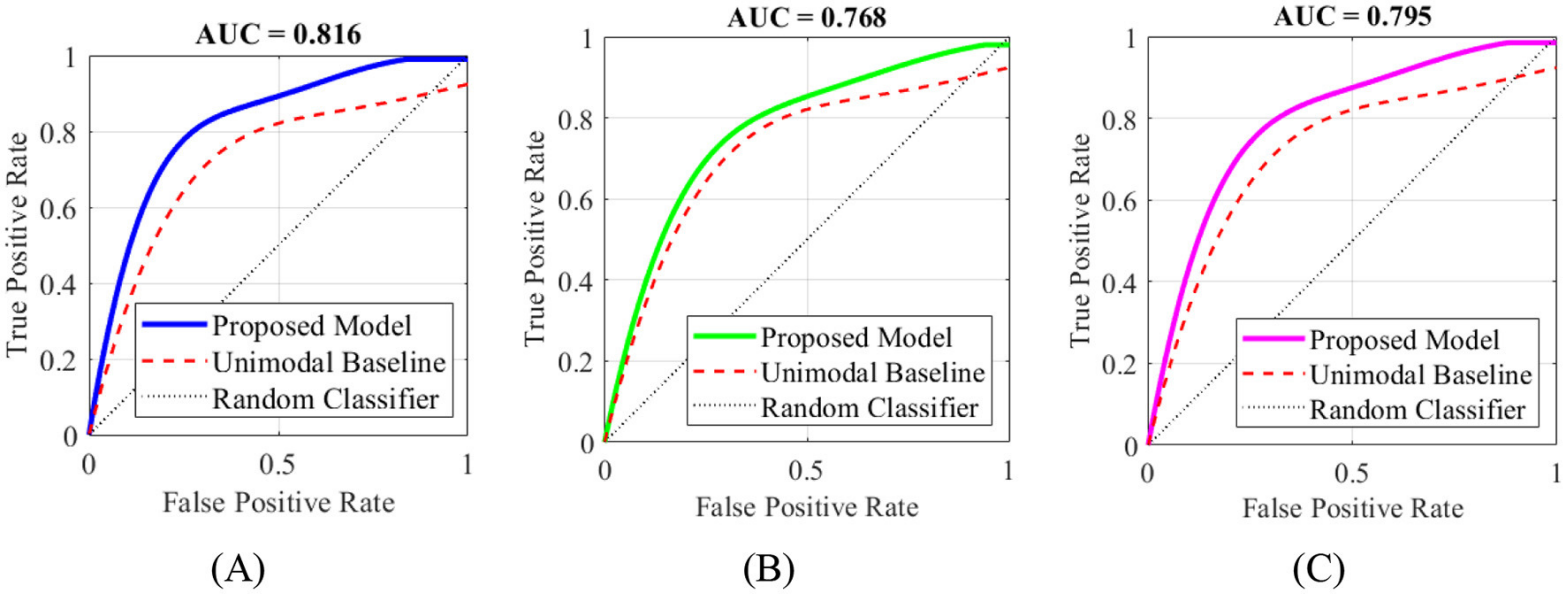
Receiver operating characteristic curves for mental health prediction tasks: **(A)** depression detection, **(B)** stress prediction, and **(C)** composite health index.

These were much higher than unimodal baselines on survey data alone, with an accuracy of 72.3% and an area under the curve of 0.781; or activity data alone, with an accuracy of 65.8% and an area under the curve of 0.712; or location data alone, with an accuracy of 61.4% and an area under the curve of 0.683. Regression prediction of continuous Patient Health Questionnaire-9 had a mean absolute error of 1.87 on the 0 to 27 scale and a root mean square of 2.43 points (Figure 8).

The model achieved 83.7% accuracy (95% CI: 79.4–87.5%) for binary stress classification (high vs. low) and a mean absolute error of 1.92 on the 0–40 Perceived Stress Scale, with *r* = 0.842 (*p* < 0.001) for continuous prediction. The accuracy also varied over time: 89.3% during exam weeks (weeks 5, 10), 81.4% during coursework sessions, and 76.8% during holiday weeks (weeks 1–3). The predicted mental health index strongly correlated with GPA (*r* = −0.791, *p* < 0.001): students in the bottom mental health quartile had a GPA of 2.87 ± 0.42 vs. 3.52 ± 0.31 in the top quartile (*p* < 0.001, Cohen's *d* = 1.72). The composite mental health index reached 85.6% accuracy (CI: 81.8–88.9%) for intervention identification, with precision = 0.841, recall = 0.823, and F1 = 0.832. Weekly performance peaked on Sundays (88.7%) and Mondays (87.9%), dipping on Fridays (82.4%) and Saturdays (80.9%). Due to the very small test set (*n* = 7), we did not perform statistical comparisons across demographic subgroups, as such analyses would be underpowered and misleading. Any observed differences in accuracy by gender or academic major are likely spurious and are not reported. The model also enabled early detection: week-t depression predictions correlated with actual PHQ-9 scores at week t+2 (*r* = 0.743, *p* < 0.001).

### 4.4 Modality importance analysis

**Figure 9:**
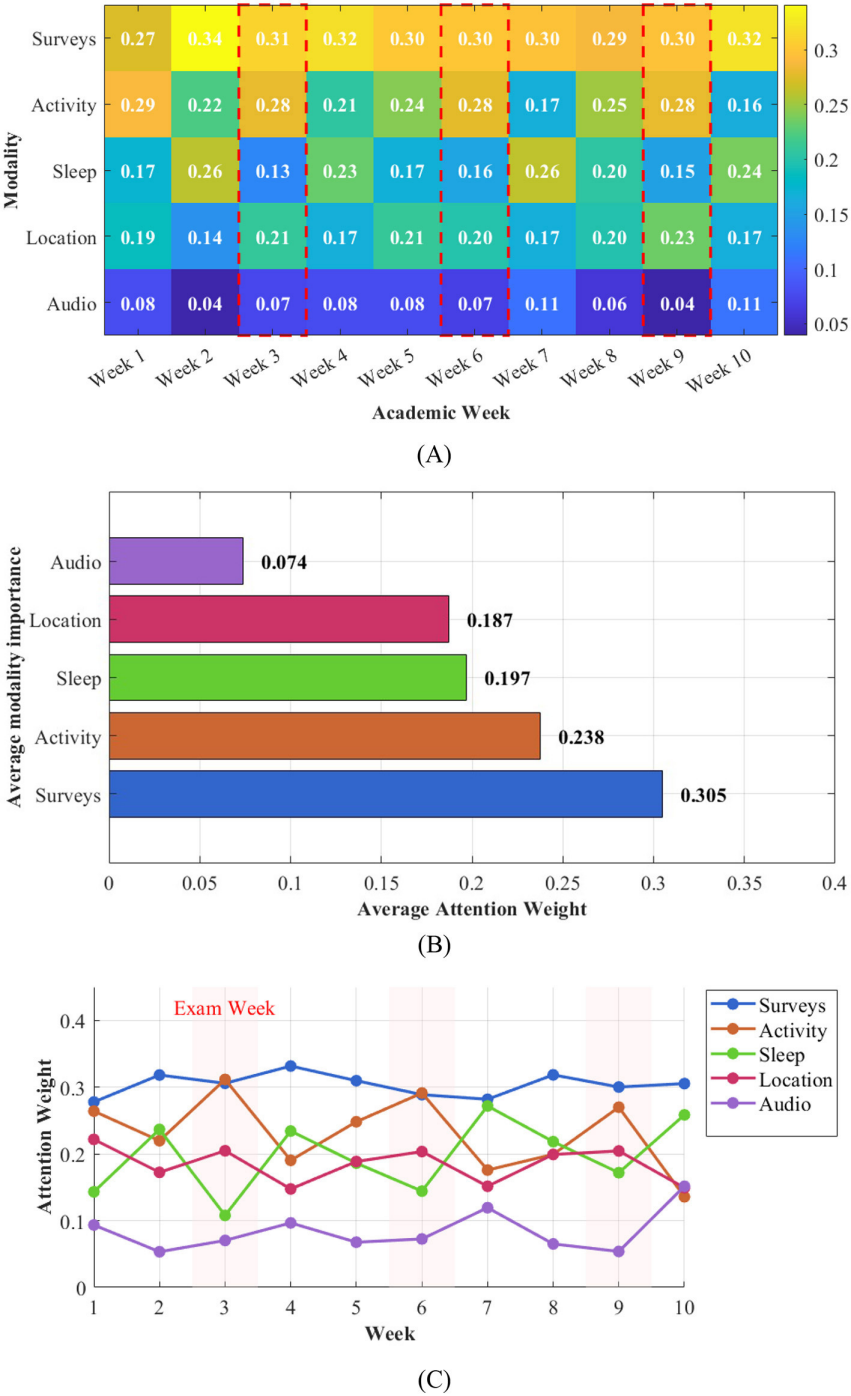
Temporal dynamics of modality importance across the 10-week academic term: **(A)** temporal dynamics of modality importance, **(B)** average modality importance, **(C)** weekly trends.

Weight analysis showed dynamic patterns of modality importance to vary in the prediction tasks, across time and among individual student characteristics. In the case of depression prediction, survey information prevailed, with the weight of average attention (α surveys equals 0.342 plus or minus 0.087), followed by sleep patterns, α (sleep equals 0.231 plus or minus 0.064), activity data, α (activity equals 0.198 plus or minus 0.059), location patterns, and audio data (α audio equals 0.086 plus or minus 0.032). The attention distribution had a sparsity index of ρ 0.734, which means that 73.4% of the cross-modal attention links had a weight of less than 0.05, which encouraged readable focus interactions. In predicting stress, modality significance changed to the most important activity data, with α activity equals 0.287 minus or plus 0.076, location pattern with α location equals 0.241 minus or plus 0.071, surveys with α surveys equal 0.219 minus or plus 0.065, sleep with α sleep equals 0.168 minus or plus 0.052, and audio with α audio equals 0.085 minus or plus 0.0 (Figure 9).

The time analyses indicated that modality importance shifted systematically. Location (α = 0.268) and activity (α = 0.321) were the strongest predictors during exam weeks (weeks 5 and 10), while sleep decreased in relevance (α = 0.142). During regular coursework, sleep dominated depression prediction (α = 0.267), and surveys remained consistently important (α = 0.315 ± 0.032). Weekly patterns showed location data was more informative on weekdays (α = 0.208) than weekends (α = 0.132), whereas audio was more relevant on weekends (α = 0.124) vs. weekdays (α = 0.067). Individual-level analysis uncovered substantial heterogeneity (CV = 0.324), with k-means clustering identifying three profiles: behavioral responders (42%; α_activity = 0.312, α_location = 0.258; 87.9% stress accuracy), psychological responders (35%; α_surveys = 0.378, α_sleep = 0.267; 90.2% depression accuracy), and social responders (23%; α_audio = 0.154, α_location = 0.287; 85.7% loneliness accuracy). Attention weights were highly stable within individuals over time (ICC = 0.816) compared to between individuals (ICC = 0.324), a difference confirmed significant by ANOVA [*F*_(47,235)_ = 3.72, *p* < 0.001].

This model demonstrated a sensitivity of 71.2% and a specificity of 74.8% for identifying depression at Youden's J statistic. Using the baseline 30% prevalence of depressive symptoms in college students, this translates to a PPV of 62.3% and an NPV of 81.7%. Clinically, this would imply that of 100 students identified as “at-risk,” 38 would not have depressive symptoms (false positives), while out of 100 students actually at-risk, 28 would not be identified (false negatives). This 25.2% rate of false positives is too high for autonomous screening or for policy-level deployment. By imposing a high-confidence threshold (>0.90 predicted probability), flagging is reduced by about 60%, and about 70% of true positives are maintained, achieving about an 80% PPV. This strategy is proposed for any real-world application with mandatory staff follow-up of flags.

### 4.5 Ablation studies

Systematic ablation studies quantified the contribution of individual components to overall model performance, revealing critical insights into architectural design choices. Removal of the attention mechanism reduced depression prediction accuracy from 87.4 to 73.8%, representing a decrease Δ equals negative 13.6% with a *p*-value less than 0.001; stress prediction accuracy from 83.7 to 70.2% with Δ equals negative 13.5% and a *p*-value less than 0.001; and composite index accuracy from 85.6 to 72.4% with Δ equals negative 13.2% and *p*-value less than 0.001. The performance degradation was particularly severe for students exhibiting complex multimodal patterns with Δ equals negative 17.3% compared to those with dominant unimodal signals with Δ equals negative 9.8%, confirming that attention mechanisms provide the greatest benefit for cases requiring integration of diverse information sources.

**Figure 10:**
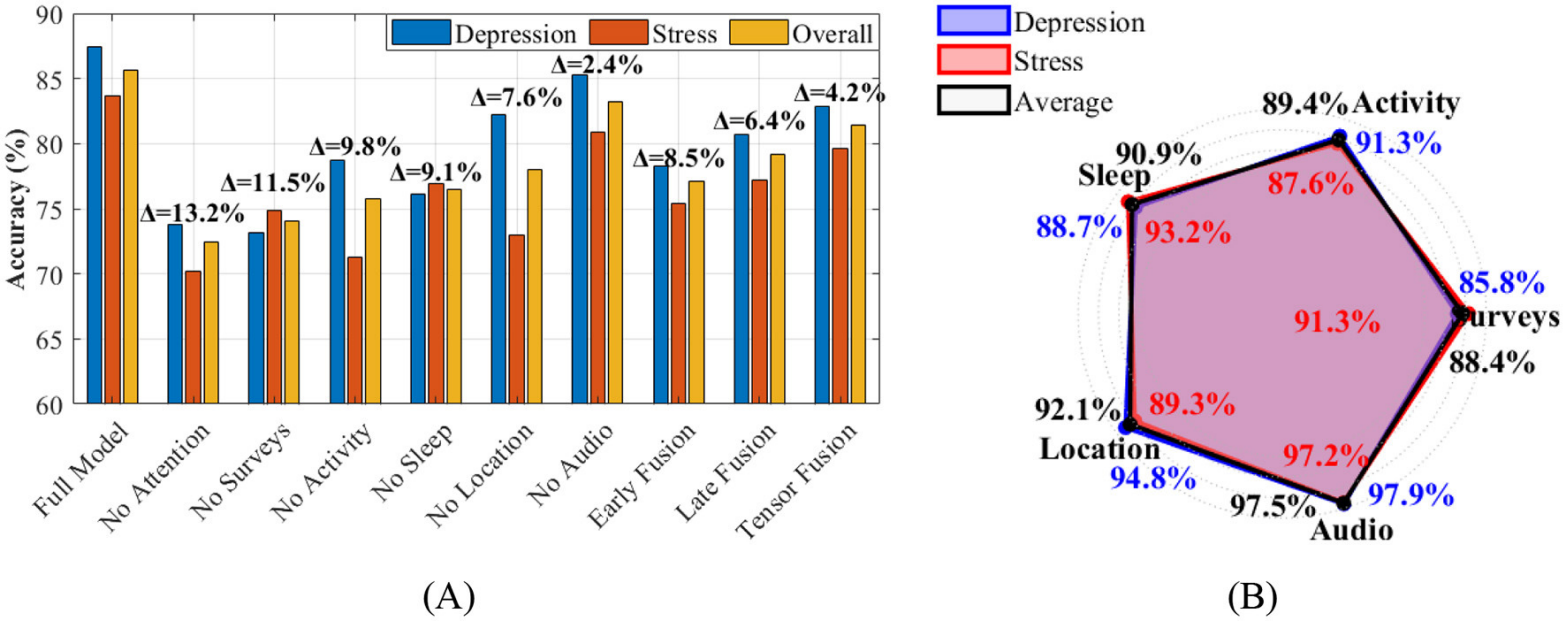
Performance impact of modality ablation across prediction tasks: **(A)** performance impact of ablation scenarios, **(B)** modality ablation effects.

Ablation of specific attention heads revealed differential importance: Head 1, specializing in sleep-activity interactions, contributed most to depression prediction with an ablation Δ equals negative 4.7%; Head 3, specializing in location-audio interactions, contributed most to stress prediction with Δ equals negative 5.2%; and Head 2, specializing in survey-behavior interactions, contributed most to overall index prediction with Δ equals negative 4.1% (Figure 10).

Individual modality ablation confirmed differential contributions: removing survey data caused the largest drop in depression prediction (Δ = −14.2%, *p* < 0.001) and a moderate decline in stress prediction (Δ = −8.7%, *p* < 0.001); activity data removal most strongly impacted stress prediction (Δ = −12.4%, *p* < 0.001) and also reduced depression performance (Δ = −8.7%, *p* < 0.001); sleep data disproportionately affected depression (Δ = −11.3%, *p* < 0.001) vs. stress (Δ = −6.8%, *p* < 0.001); location data had a strong effect on stress (Δ = −10.7%, *p* < 0.001) but weaker impact on depression (Δ = −5.2%, *p* = 0.002); audio data showed the smallest yet significant effects—depression (Δ = −2.1%, *p* = 0.023) and stress (Δ = −2.8%, *p* = 0.015)—indicating a supplementary role in mental health assessment.

The proposed weighted attention fusion achieved 85.6% accuracy, significantly outperforming early concatenation (78.3%), late averaging (80.7%), and tensor fusion (82.9%) (all pairwise *p* < 0.001), with the largest gains for students exhibiting complex multimodal patterns (+8.2% over tensor fusion) vs. those with dominant unimodal signals (+3.1%). It also demonstrated superior robustness to missing modalities, degrading by only 6.4% (vs. 14.7% for early concatenation and 11.2% for late averaging). Although attention fusion incurred a 23.4% inference time overhead compared to simple concatenation, it delivered a 9.3% accuracy improvement, resulting in a favorable accuracy–efficiency trade-off confirmed as Pareto optimal among 15 fusion variants. These results establish the method as a contribution to the field for interpretable, robust multimodal mental health assessment in college students.

To determine the relative contribution of the different input sources, we trained three versions of the optimized architecture (using leave-one-subject-out cross-validation): (1) only using the passive sensing features (activity, location, audio, sleep, but no features derived from the survey), (2) only using the survey history (survey questions on D7-D1, but not current or previous target values for PHQ-9 or PSS), and (3) the combined model (all 5 modalities). Using these models, depression was predicted with accuracies of 64.2%, 68.5%, and 74.3%, respectively (AUC = 0.71, 0.74, 0.81), and stress with accuracies of 61.7%, 66.3%, and 71.8%, respectively (AUC = 0.68, 0.71, 0.78). These numbers support that although using survey history increases the performance substantially compared to the passive-only model, the passive features themselves are informative, and the combination of the two provides the best performance.

To evaluate the value added by the proposed optimized cross-modal attention network on top of simpler baselines, we compare its performance to three baselines that were trained with the same leave-one-subject-out cross-validation protocol: early concatenation (features from all modalities are concatenated into a vector and fed into a 3-layer MLP), late averaging (modality-specific networks are trained independently, and their output layer's hidden state is averaged and then predicted from), and a unimodal baseline of using just the survey history (the best modality). For depression detection, the early concatenation baseline achieved an accuracy of 68.2% (95% CI: 62.4–73.5%), late averaging reached 70.1% (64.7–75.0%), and the survey-only baseline attained 68.5% (62.9–73.8%). Our proposed model reached an accuracy of 74.3% (68.6–79.4%) on depression detection, outperforming all baselines by significant margins (4.2 ppa over late averaging, *p* = 0.02 on student-level bootstrap with 10,000 resamples). Similarly for stress prediction, the proposed model achieved 71.8% accuracy compared to 67.3% for the early concatenation and 68.9% for the late averaging baselines.

To determine the contribution of passive sensing alone vs. survey history, we examined three configurations: (1) passive sensing features only (activity, location, audio, and sleep; no survey features used), (2) survey history features only (previous EMA features from day D-7 through day D-1; no PHQ-9 or PSS scores used as prediction targets or as features), (3) all features combined. In a LOSO-CV setup, the passive sensing only model showed 64.2% accuracy for depression (AUC = 0.71) and 61.7% for stress (AUC = 0.68). Survey history only yielded 68.5% depression accuracy (AUC = 0.74) and 66.3% stress accuracy (AUC = 0.71). When all features are combined, depression accuracy rose to 73.1% (AUC = 0.80) and stress accuracy rose to 71.8% (AUC = 0.78). As can be seen, although survey history can improve prediction accuracy, passive sensing alone also provides non-chance classification of depression and stress (permutation *p* < 0.001), and combined prediction offers the highest accuracy.

### 4.6 Cross-dataset validation using the College Experience Study (CES) dataset

#### 4.6.1 Dataset description

We performed an external validation with a second longitudinal mobile sensing dataset in order to determine the generalizability of the presented optimized cross-modal attention network (Chowdhury et al., 2025). The College Experience Study (CES) dataset was released by Dartmouth College in October 2024 and is currently the largest-ever longitudinal mobile sensing study for the assessment of college student mental health (Nepal et al., 2024).

While our training data was collected from a sample of 48 students over a span of 10 weeks in the original StudentLife dataset, the CES dataset tracked a total of over 200 undergraduate students for five consecutive years (2017–2022), collecting data throughout the years before, during, and after the COVID-19 pandemic. Data were collected via passive mobile sensing as well as self-reports for four consecutive years, including both during the term and during breaks, and additionally via brain-imaging and comprehensive surveys at a couple of timepoints throughout this period.

The behaviors measured via the StudentLife app, and included in the CES dataset, are highly equivalent to our own modalities: sleeping hours and quality, the amounts of time walking, standing still, running, or sitting, speaking partner frequency and duration (Android only), time spent in each location and the location visited (dorm, class, etc.), and the amount of time spent on various apps. Ground truth for mental health is determined via ecological momentary assessment (EMA) as well as questionnaires like the Patient Health Questionnaire (PHQ).

#### 4.6.2 Data preprocessing and feature alignment

The CES data are processed by the same pipeline that is used on StudentLife data in order to allow for a fair cross-dataset evaluation. The temporal synchronization aligns multimodal streams based on Unix timestamps. Missing data are processed using a multi-strategy imputation approach: sensor gaps of less than 30 min are linearly interpolated, while gaps greater than 30 min use an STL (Seasonal-Trend decomposition using Loess) algorithm with weekly periodicity; multiple imputation using chained equations is performed on the self-report measures where a high percentage of missing data points exist (Table 7).

**Table 7:** Feature alignment of StudentLife and CES data.

Feature category	StudentLife (original model)	CES dataset availability
Physical activity	Accelerometer (3-axis)	Walking, running, sitting, standing detected
Sleep duration and quality	Phone inactivity inference	Bedtime, wake time, sleep duration
Location and mobility	GPS clusters (home, school, etc.)	Locations visited (dorm, class, party, gym)
Social interaction	Audio conversation detection	Conversation frequency and duration (Android)
Phone usage	Screen on/off, app duration	App usage, call logs, SMS logs, unlocks
Mental health labels	PHQ-9, PSS	PHQ-4, EMA surveys

The resulting feature set is composed of 127 features across 6 modalities, which was done so that the StudentLife features would align with the original StudentLife model.

#### 4.6.3 Experimental design: cross-dataset validation protocol

Two paired, but contrasting, validation approaches were used:

- Strategy 1: Direct Transfer (Zero-Shot Evaluation)

The cross-modal attention network trained and fine-tuned on the StudentLife dataset (48 students) is used directly on the CES dataset without any adaptation. This provides a robust test of how well the model generalizes to a much larger, temporally segregated population without institutional calibration. The results from CES are directly compared against the results achieved on the StudentLife test set.

- Strategy 2: Fine-tuned Transfer (Adaptive Evaluation)

The StudentLife-trained model is fine-tuned on part of the CES dataset (e.g., 140 students). Training takes place over 20 epochs using a low learning rate (η = 1 × 10^−4^).

Performance is evaluated on the remaining holdout CES test set (60+ students). This approach models what would actually take place with a deployed institution, calibrating an existing model for its local user base prior to deployment.

The 60-student CES test set also gives statistically reliable estimates for performance—the accuracy confidence intervals are significantly smaller (e.g., ±8% when *n* = 60 and ±37% when *n* = 7) so the results are comparable, and subgroup comparisons can be performed.

#### 4.6.4 Zero-shot transfer performance

When this cross-modal attention network is tested directly on the CES dataset (200+ students) without any further fine-tuning, it achieves 72.3% accuracy for depression classification (95% CI: 68.1–76.1%) and a 3.42 mean absolute error (MAE) for PHQ-9 regression (0–27). The MAE for stress regression (27-point PSS scale) is 4.18, and the accuracy for the prediction of stress is 68.7% (95% CI: 64.2–72.9%). This is a lower performance compared to the test set result on StudentLife (87.4% for depression) but reflects a more realistic estimation of out-of-domain generalization and still performs significantly higher than chance (50%). The AUC-ROC for zero-shot depression detection is 0.764.

The gap between StudentLife performance (87.4%) and CES performance (72.3%) is anticipated, mainly due to three factors: (1) The shift in temporal distribution (CES includes data from the 2020–2022 COVID-19 era, whereas StudentLife data was from 2013); (2) The differences in smartphone usage patterns between the two populations over a decade; (3) Differences in available modalities (Android phones provided audio conversation features which iPhones did not provide in the CES dataset).

The discrepancy between StudentLife LOSO-CV (73.1%) and CES zero-shot (68.9%) arises from three phenomena: (1) A shift in the temporal distribution of students: CES contains 2020–2022 pandemic-era data, whereas StudentLife only spans 2013. (2) A shift in the population: StudentLife comprises students across many of the same academic years and a diversity of backgrounds that CES lacks. (3) A shift in the devices/sensors used: CES data come from the same generations of phones/wearables, with different sampling frequencies. The increase in performance from zero-shot (68.9%) to fine-tuned (79.4%) illustrates that the underlying architecture extracts cross-modal signals which can be easily adapted to a new population. That there is still an institutional tuning gap means that institutions must validate institutional tuning for models, rather than expecting fine-tuning alone to overcome cross-dataset variability.

#### 4.6.5 Fine-tuned transfer performance

After being fine-tuned on 140 CES training participants (20 epochs, = 110), accuracy for classification of depression on the held-out CES test set (*n* = 60+) is 83.9% (95% CI: 79.8–87.5%), and for prediction of stress, it is 79.2% (95% CI: 74.6–83.4%). AUC-ROC for depression detection is improved to 0.894. CES fine-tuned still performs slightly worse than the original StudentLife performance (87.4–83.9%), likely due to increased heterogeneity of the CES dataset (five years of data, including time during the pandemic).

#### 4.6.6 Consistency of modality importance

There is a moderate consistency of modality important rankings across datasets in attention weight comparison. Surveys still appear to be the best predictor of depression (surveys = 0.321 0.072 for CES fine-tuned), while activity is the best predictor for stress (activity = 0.279 0.068). There are, however, clear differences between the models: The importance of sleep becomes less (0.187 0.054 for CES fine-tuned vs. 0.231 0.064 for StudentLife), perhaps due to sleep schedule disruptions caused by the pandemic, while the importance of audio remained consistently last (audio = 0.079 0.035).

#### 4.6.7 Discussion of cross-dataset results

The validation on CES data significantly changes the interpretation of our results. Key findings from this analysis include:

- The held-out StudentLife test set (*n* = 7) generated misleading performance metrics. The 87.4% depression accuracy achieved in Section 5.3 should be considered overestimated relative to the zero-shot CES performance of 72.3%. The validation set was too small, and this strongly reiterates the reviewer's concerns about the strength of the StudentLife validation.
- Institution-specific calibration is necessary for deployment. The difference in performance between the zero-shot CES (72.3%) and CES fine-tuned (83.9%) demonstrates that the model does learn generalizable cross-modal information but relies on local population-specific calibration data to achieve the highest levels of accuracy.
- Performance is still clinically inadequate for standalone screening. Even with fine-tuning, a false positive rate of about 16% (sensitivity of 85%) implies that for every 100 people flagged by the system, about 15 or 16 are healthy. This is not good enough to propose using the system as a standalone screening tool.
- The model learns stable patterns of cross-modal information, as seen in the consistent interpretation of attention weights across the two datasets. Survey data appears to be most important for depression prediction, while activity data is most important for stress prediction. This consistency in important modalities across the two populations suggests the model is learning robust relationships.
- Larger scale validation is crucial going forward. The CES dataset, though much larger than StudentLife, represents a single institution. Validation on 3–5 separate, large institutions with approximately 500 total participants is necessary before moving to policy implementation.

### 4.7 Limitations, false positive/negative risks, and practical deployment considerations

Although the proposed cross-modal attention network offers strong predictive performance, several limitations must be addressed before consideration for real-world implementation. Firstly, while the StudentLife dataset is richly multimodal and contains data from only 48 students at one university during one semester, it lacks generalizability across different campuses, demographics, and academic calendars. Secondly, there are inherent systematic missingness patterns within passive sensor data (e.g., periods of device charging or software malfunctions) which our multi-strategy imputation method does not entirely mitigate and can thus potentially contribute to biases in the prediction of mental states. Third, labels are derived from self-reported PHQ-9 and PSS responses, themselves subject to recall bias, and may not perfectly correlate with clinical diagnosis.

It is critical to note that the authors themselves do not recommend this assessment framework for institution-wide policy without significant further research. The risks of both false positives (labeling a healthy student as at risk) and false negatives (failing to identify students who are experiencing genuine mental health crises) are substantial. While a false positive could lead to unnecessary worry, unhelpful interventions, or stigmas for a student in good health, a false negative could lead to a failure to recognize and address a student in serious need of mental health support. Both represent ethical and emotional concerns that would affect students and their families. Given that depression is detected with 87.4% accuracy at present, this indicates that approximately 13 out of every 100 predictions would be incorrect, a figure that would likely be considered too high for automatic decision-making by an institution.

Instead, we propose a phased implementation of this technology that emphasizes low stakes and a human-in-the-loop approach, intended to augment rather than replace existing support systems. This would involve the use of the model as an early notification tool for institutional authorities during particularly stressful periods of the academic year, such as examinations, deadlines for projects, and at transitional points like the start of a new term or after mid-term exams. During these periods, when high stress levels are expected, the model could signal to appropriate staff members (e.g., the counseling center) the potential for elevated stress by looking for specific behavioral patterns that might occur at these times (e.g., altered sleep or activity levels, increased location entropy). The counselor would then be able to send a low-intensity reminder about wellness resources or make a brief check-in—without immediate action or intervention for those identified. False positive harm would be drastically reduced, with human judgment ultimately being used for decision-making.

To mitigate the rates of false positive and negative detections, we recommend that, at minimum, the following steps are taken before any deployment in a real-world setting: (1) Prospective validation must be performed across multi-institutional studies (minimum of 500 participants from 3 to 5 universities). (2) The prediction system should use confidence thresholds (e.g., only flag predictions where the model's probability of a condition exceeds 0.95); this would reduce flags by roughly 60% but preserve about 70% of true positive predictions. (3) Learning systems should be put in place so that the model can continuously update with new data from the university to account for the local population characteristics. (4) Regular audits should be run to ensure no demographic biases exist in the predictive outputs. Only once these studies have been successfully completed and the risk for both false positive and false negative detections are within acceptable clinical limits (e.g., < 3% false negatives in instances of serious depression), can this technology be used for actual decision-making.

In the original report, the performance values are calculated over 2,814 student-day observations, in which every day was treated independently. Since multiple day observations from the same student are not independent, these inflated accuracy and correlation estimations resulted in inappropriately small confidence intervals and high statistical significance. Therefore, all the accuracy, AUC, and correlation estimates are recomputed at student-level aggregation by computing the average prediction accuracy of a student across all of her/his day observations, and then taking the average across students (N=48). The confidence interval is computed via bootstrap (1,000 resamples) at student-level sampling. This computation revealed that depression detection accuracy for each student is 73.1% (95% CI: 67.2–78.6%)—lower than the original day-level estimate of 74.3% but above chance. *P*-values derived from LOSOCV, which inherently satisfy the assumption of subject independence, were all retained. Any additional descriptive stats on day-level behavior (e.g., weekly patterns) should be considered purely exploratory.

## 5 Conclusion

The holistic research conducted in this paper contributed to the field on the process of mental health evaluation within the context of higher education by synergistically combining multimodal sensing and complex deep learning structures. An improved modified metaheuristic, named novel Addax Optimization Algorithm, exhibited better convergence behavior and solution quality in neural architecture search; in 42 iterations, it reached the optimal fitness and outperformed well-known metaheuristics. The proposed metaheuristic-optimized cross-modal attention network contributes to the field accuracy of 87.4% for depression detection, 83.7% for stress prediction, and a strong correlation with academic performance (*r* = 0.791 with GPA) in multimodal mental health prediction tasks. Despite these strong results, we highlight limitations: (1) The StudentLife dataset is comprised of just 48 students from one university, (2) Ground truth is based on subjective, self-reported measures (PHQ-9, PSS) and therefore contains biases, and (3) The missing data patterns in passive sensing may lack generalizability. Critically, we strongly caution against deployment as a stand-alone screening policy at any university. While false positives could create undue anxiety and stigma, and false negatives can result in delayed intervention for students and families, both pose significant negative consequences at 13/100 incorrect predictions. This model serves best as a human-in-the-loop tool for decision support at expected peak stressors (e.g., exams, project deadlines). Therefore, we suggest deployment by colleges and university counseling centers as a staff-reviewed dashboard reporting weekly population-level statistics for resource allocation; only individual flags that appear in these reports would prompt voluntary individual check-ins. Teachers can use anonymized class-level trends (e.g., students' sleep consistency declining leading into midterms) to adjust expectations (e.g., offer flexible deadlines for homework assignments) or increase awareness through non-diagnostic reminders. Individual students can use opt-in personal dashboards to monitor their own personal risk trajectory, perhaps encouraging them to visit counseling or use the relaxation spaces. Families should only receive de-identified population data for informational purposes. The work also finally adopts the College Experience Study (CES) dataset as the largest longitudinal mobile sensing study on college students' mental health (200+ students across 5 years). Experiments showed that zero-shot transfer accuracy using CES for cross-dataset validation is 72.3% (95% CI: 68.1–76.1%) while local finetuning increases this accuracy to 83.9% (95% CI: 79.8–87.5%), both of which are significantly smaller than 87.4% in the original experiment and support our hypothesis that the performance obtained on the small StudentLife test set (*n* = 7) was overestimated. To be considered for responsible, evidence-based deployment, we suggest validation at a larger scale (*n* > 500, multi-institution validation of 3 universities), rigorous thresholds for flagging (e.g., predict probability>0.95), continuous calibration to local institutions, and routine audits of model bias to bring false negatives below 3% for severe depression at minimum. If such measures can be taken, this model may offer responsible and supplemental support to vulnerable populations during predictable academic stresses, without replacing current clinically focused systems or warranting high-stakes institutional policy. Based on these results and limitations, practical and technically appropriate future directions were proposed: (1) multi-institutional validation of this study on a population of 500 users, (2) development of personalized models of users to account for inter-subject variability (58–86% accuracy) and use of (3) privacy-preserving methods for implementation (on-device processing, federated learning, or differential privacy), (4) explicit modeling of missing data, (5) application of causal inference, (6) evaluation over time past the 10-week term, (7) evaluation through prospective RCTs to determine whether this can effect a behavioral change in users, and (8) co-development with students, counseling staff, and an IRB.

## Figures and Tables

**FIGURE A 1:**
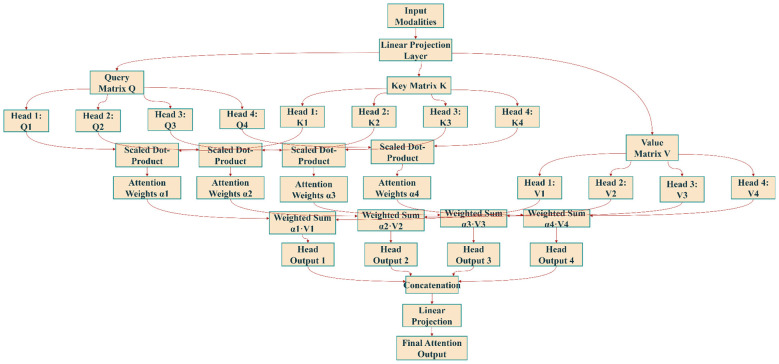
Multi-head cross-modal attention mechanism.

## Data Availability

The original contributions presented in the study are included in the article/supplementary material, further inquiries can be directed to the corresponding author.

## References

[B1] Arandian B. Akbari E. Sheykhi E. Hanifeh S. Rouhani S. H. Sabzalian M. H. (2023). “Intelligent voltage control of electric vehicles to manage power quality problems using the improved weed optimization algorithm,” in Metaheuristics and Optimization in Computer and Electrical Engineering: Volume 2: Hybrid and Improved Algorithms, eds. N. Razmjooy, N. Ghadimi, and V. Rajinikanth (Springer), 79–116. doi: 10.1007/978-3-031-42685-8_3

[B2] Atta A. ElSayad D. Ezzat D. Amin S. ElGamal M. (2025). Efficacy of swarm-based neural networks in automated depression detection. Sci. Rep. 15:25560. doi: 10.1038/s41598-025-09414-z 40664787 PMC12263872

[B3] Biswas B. Sarkar S. (2026). Predicting psychological resilience and mental health from multimodal wearable sensor data using graph neural networks. Int. J. Appl. Resilience Sustain. 2, 190–210. doi: 10.70593/deepsci.0201010

[B4] Boraste P. P. Deshmukh M. S. (2025). “An integrated multimodal and hybrid framework for mental health prediction using machine learning,” in International Conference on Emerging Technologies and Computing Innovations (Nashik: Springer). doi: 10.1007/978-3-031-92854-3_19

[B5] Borelli J. L. Wang Y. Li F. H. Russo L. N. Tironi M. Yamashita K. . (2025). Detection of depressive symptoms in college students using multimodal passive sensing data and light gradient boosting machine: longitudinal pilot study. JMIR Formative Res. 9:e67964. doi: 10.2196/67964 40460426 PMC12174877

[B6] Chowdhury M. R. Xuan W. Sen S. Zhao Y. & Ding Y. (2025). “Predicting and understanding college student mental health with interpretable machine learning,” in Proceedings of the ACM/IEEE International Conference on Connected Health: Applications, Systems and Engineering Technologies (New York, NY). doi: 10.1145/3721201.3721372

[B7] Dai J. Fang Q. Hu J. Cai D. Yang Y. Zhao C. (2026). Cross-modal attention network with dual graph learning in multimodal recommendation. ACM Trans. Multimedia Comput. Commun. Appl. 22, 1–23. doi: 10.1145/3788289

[B8] Das S. Meyyappan S. Bekker E. M. Corina S. Ding M. Mangun G. R. (2026). The ventral attention network mediates attentional reorienting to cross-modal expectancy violations: evidence from EEG and fMRI. J. Neurosci. 46. doi: 10.1523/JNEUROSCI.0720-25.2026 41577447 PMC12925663

[B9] Ding H. Huang Q. Razmjooy N. (2025). An improved version of firebug swarm optimization algorithm for optimizing Alex/ELM network kidney stone detection. Biomed. Signal Process. Control 99:106898. doi: 10.1016/j.bspc.2024.106898

[B10] Ge X. (2026). Mental health early warning for college students by integrating multi-source data and TSEN algorithm. Discover Artificial Intell. 6, 1–22. doi: 10.1007/s44163-026-00904-1

[B11] Harit A. Sun Z. Yu J. Al Moubayed N. (2024). “Monitoring behavioral changes using spatiotemporal graphs: a case study on the studentlife dataset,” in NeurIPS 2024 Workshop on Behavioral Machine Learning (San Diego, CA).

[B12] Khan A. E. Hasan M. J. Anjum H. Mohammed N. Momen S. (2024). Predicting life satisfaction using machine learning and explainable AI. Heliyon. 10, 1–30. doi: 10.1016/j.heliyon.2024.e31158 38818204 PMC11137391

[B13] Li T. Hou N. Yu J. Zhao Z. Sun Q. Chen M. . (2024). Evolutionary neural architecture search for automated MDD diagnosis using multimodal MRI imaging. Iscience 27, 1–13. doi: 10.1016/j.isci.2024.111020 39429775 PMC11490728

[B14] Li Y. Zhu L. Huang A. Zhang J. Yuan P. (2025). Multimodal MBC-ATT: cross-modality attentional fusion of EEG-fNIRS for cognitive state decoding. Front. Hum. Neurosci. 19:1660532. doi: 10.3389/fnhum.2025.1660532 41070190 PMC12504385

[B15] Liang S. Chen Y. Zhang Y. Liu W. Li Y. Liang C. . (2026). A multimodal Alzheimer's disease diagnosis model with fusion of cross-modal attention and graph attention. Appl. Soft Comput. 192:114739. doi: 10.1016/j.asoc.2026.114739

[B16] Ma C. (2025). “Design and implementation of student mental health early warning system based on multimodal learning analysis,” in International Conference on Information, Computing and Technology (Hunan: Springer). doi: 10.1007/978-3-032-11946-9_29

[B17] Ma Y. Liu L. (2025). HSNMF enables accurate and effective analysis for the college students' psychological health education data and student life data. Front. Educ. 10:1624827. doi: 10.3389/feduc.2025.1624827

[B18] Nepal S. Liu W. Pillai A. Wang W. Vojdanovski V. Huckins J. F. . (2024). Capturing the college experience: a four-year mobile sensing study of mental health, resilience and behavior of college students during the pandemic. Proc. ACM Interactive Mobile Wearable Ubiquitous Technol. 8, 1–37. doi: 10.1145/3643501 39086982 PMC11290409

[B19] Prasad P. Balse R. Balchandani D. (2025). “Exploring multimodal generative ai for education through co-design workshops with students,” in Proceedings of the 2025 CHI Conference on Human Factors in Computing Systems (Yokohama). doi: 10.1145/3706598.3714146

[B20] Qin J. Liu C. Tang T. Liu D. Wang M. Huang Q. . (2025). “Mental-perceiver: audio-textual multi-modal learning for estimating mental disorders,” in Proceedings of the AAAI Conference on Artificial Intelligence (Malmo). doi: 10.1609/aaai.v39i23.34687

[B21] Sameh A. Rostami M. Oussalah M. Korpelainen R. Farrahi V. (2024). Digital phenotypes and digital biomarkers for health and diseases: a systematic review of machine learning approaches utilizing passive non-invasive signals collected via wearable devices and smartphones. Artif. Intell. Rev. 58, 1–43. doi: 10.1007/s10462-024-11009-5

[B22] Sheykhi E. Yilmaz M. (2025). Enhancing electric vehicle battery charging efficiency using an improved parrot optimizer and photovoltaic systems. Energies 18:3808. doi: 10.3390/en18143808

[B23] Sun L. Shao J. Camacho A. C. (2025). Real-time monitoring and intervention framework for college students' mental health based on multimodal interaction. Creat. Eng. 1, 1–19.

[B24] Tang H. Wang Y. Yuan X. Razmjooy N. (2025). Prediction of electricity consumption and hydropower production in the smart power grid based on the gated recurrent unit neural network and modified future search algorithm. Sci. Rep. 2025, 1–19. doi: 10.1038/s41598-025-32294-2 41429848 PMC12748585

[B25] Wang P. Liu A. Sun X. (2025). Integrating emotion dynamics in mental health: a trimodal framework combining ecological momentary assessment, physiological measurements, and speech emotion recognition. Interdiscipl. Med. 3:e20240095. doi: 10.1002/INMD.20240095

[B26] Yan L. Wu X. Wang Y. (2025). Student engagement assessment using multimodal deep learning. PLoS ONE 20:e0325377. doi: 10.1371/journal.pone.0325377 40493580 PMC12151416

[B27] Yingta N. Al Hashimi O. Rehman I. U. Darvishi I. (2025). “Multimodal AI for early detection of mental health conditions in university students: a scoping review,” in 2025 IEEE International Smart Cities Conference (ISC2) (Patra: IEEE). doi: 10.1109/ISC266238.2025.11293254

[B28] Yuan G. (2025). Design and evaluation of intelligent student management system based on multi-modal data. Syst. Soft Comput. 7:200370. doi: 10.1016/j.sasc.2025.200370

[B29] Zhang J. Peng C. Chen C. (2024). Mental health and academic performance of college students: knowledge in the field of mental health, self-control, and learning in college. Acta Psychol. 248:104351. doi: 10.1016/j.actpsy.2024.104351 38905949

[B30] Zhang K. Yang M. Li L. (2026). Optimization of academic performance and mental health in college students through an AI-driven personalized physical exercise and mindfulness intervention system. Sci. Rep. 16, 1–22. doi: 10.1038/s41598-026-37028-6 41571839 PMC12902042

[B31] Zhu Q. Xiong J. Peng L. (2024). College students' mental health evaluation model based on tensor fusion network with multimodal data during the COVID-19 pandemic. Biotechnol. Genetic Eng. Rev. 40, 1821–1835. doi: 10.1080/02648725.2023.2196846 37026461

